# Evolutionary History and Attenuation of Myxoma Virus on Two Continents

**DOI:** 10.1371/journal.ppat.1002950

**Published:** 2012-10-04

**Authors:** Peter J. Kerr, Elodie Ghedin, Jay V. DePasse, Adam Fitch, Isabella M. Cattadori, Peter J. Hudson, David C. Tscharke, Andrew F. Read, Edward C. Holmes

**Affiliations:** 1 CSIRO Ecosystem Sciences, Canberra, Australian Capital Territory, Australia; 2 Center for Vaccine Research, Department of Computational and Systems Biology, University of Pittsburgh School of Medicine, Pittsburgh, Pennsylvania, United States of America; 3 Center for Infectious Disease Dynamics, Department of Biology, The Pennsylvania State University, University Park, Pennsylvania, United States of America; 4 Research School of Biology, The Australian National University, Canberra, Australian Capital Territory, Australia; 5 Fogarty International Center, National Institutes of Health, Bethesda, Maryland, United States of America; Emory University, United States of America

## Abstract

The attenuation of myxoma virus (MYXV) following its introduction as a biological control into the European rabbit populations of Australia and Europe is the canonical study of the evolution of virulence. However, the evolutionary genetics of this profound change in host-pathogen relationship is unknown. We describe the genome-scale evolution of MYXV covering a range of virulence grades sampled over 49 years from the parallel Australian and European epidemics, including the high-virulence progenitor strains released in the early 1950s. MYXV evolved rapidly over the sampling period, exhibiting one of the highest nucleotide substitution rates ever reported for a double-stranded DNA virus, and indicative of a relatively high mutation rate and/or a continually changing selective environment. Our comparative sequence data reveal that changes in virulence involved multiple genes, likely losses of gene function due to insertion-deletion events, and no mutations common to specific virulence grades. Hence, despite the similarity in selection pressures there are multiple genetic routes to attain either highly virulent or attenuated phenotypes in MYXV, resulting in convergence for phenotype but not genotype.

## Author Summary

The text-book example of the evolution of virulence is the attenuation of myxoma virus (MYXV) following its introduction as a biological control into the European rabbit populations of Australia and Europe in the 1950s. However, the key work on this topic, most notably by Frank Fenner and his colleagues, occurred before the availability of genome sequence data. The evolutionary genetic basis to the major changes in virulence in both the Australian and European epidemics is therefore largely unknown. We provide, for the first time, key details on the genome-wide changes that underpin this landmark example of pathogen emergence and virulence evolution. By sequencing and comparing MYXV genomes, including the original strains released in the 1950s, we show that (i) MYXV evolved rapidly in both Australia and Europe, producing one of the highest rates of evolutionary change ever recorded for a DNA virus, (ii) that changes in virulence were caused by mutations in multiple genes, often involving losses of gene function due to insertions and deletions, and that (iii) strains of the same virulence were defined by different mutations, such that both attenuated and virulent MYXV strains are produced by a variety genetic pathways, and generating convergent evolution for phenotype but not genotype.

## Introduction

The classic model of pathogen evolution following a species jump is the introduction of the lethal myxoma virus (*Poxviridae*, genus *Leporipoxvirus*) into the European rabbit (*Oryctolagus cuniculus*) populations of Australia as a biological control [1]. MYXV causes a largely innocuous cutaneous fibroma in its natural host, the South American tapeti (*Sylvilagus brasiliensis*). However, in European rabbits the same virus causes the systemic lethal disease myxomatosis. A strain of this highly virulent virus – Standard Laboratory Strain (SLS) – was successfully introduced into wild European rabbits in Australia in 1950 and over the next five years spread across the entire continental range of the rabbit. With outstanding foresight, Fenner and colleagues tracked the phenotypic evolution of virulence in the Australian epidemic by testing the lethality of viral isolates taken from the field over the subsequent decades in laboratory rabbits (of the same species as those infected in the wild).

SLS had a case fatality rate (CFR) estimated at 99.8% [1]. However, within two years of the introduction of SLS, and despite the ongoing release of virulent viruses, slightly attenuated MYXV strains came to dominate field populations. Although they still killed 90–99% of infected rabbits, these lower virulence strains allowed infected rabbits to survive for longer, increasing the probability of transmission from skin lesions by mosquito vectors [2], [3]. MYXV virus does not replicate in the vector [2], [4], so that transmission is dependent on high titres in cutaneous lesions (≥10^7^ infectious particles/g) and the duration of survival of the infected rabbit. Highly attenuated viruses could be quickly controlled by the rabbit immune system and so tended to have relatively low transmissibility [2]. For the next 30 years the majority of MYXV were of intermediate virulence with CFRs of 70–95% in laboratory rabbits [5]. At the same time, natural selection acted on the wild rabbit population, resulting in the emergence of animals resistant to myxomatosis [6], [7], likely through an enhanced innate immune response [8], [9]. This effectively reduced the virulence of field strains of MYXV in wild compared to laboratory rabbits. Uniquely, this evolutionary experiment was repeated in Europe following the release of a separate South American strain of MYXV (Lausanne strain; Lu) into France in 1952. The evolutionary outcomes were strikingly similar to those in Australia, with the emergence of attenuated virus strains and selection for resistant rabbits [1].

These remarkable natural experiments in biological control have become the bedrock of the mathematical theory of virulence evolution [10]–[13], revealing much about the relationship between virulence and transmissibility. The hypothesis is that a reduction in virulence was selectively favoured because, by killing hosts so rapidly, highly virulent viruses had shorter infectious periods and hence lower fitness. The emergence of attenuated viruses may also have aided the selection of resistant rabbits [1], [7], in turn driving the evolution of more virulent MYXV in subsequent decades in an archetypal host-pathogen arms race [14], [15].

Despite the canonical position of MYXV in studies of virulence evolution, the critical body of work occurred in the pre-genomic era. Today, we know that at least 20 genes encoding proteins with immune evasion or host range functions have a demonstrated role in MYXV virulence based on gene knock-out studies, and a further 20 proteins are predicted to have similar functions [16], [17]. However, both the pattern of molecular evolution and the genetic basis to the changes in virulence in both the Australian and European epidemics is largely unknown. Here we use comparative genomics to illuminate these uniquely important evolutionary events.

## Results

### Evolutionary history and dynamics of MYXV

The genome of the Lu strain of MYXV comprises 161.8 kb of double-strand (ds) DNA with terminal inverted repeats (TIRs) of 11,577 bp. MYXV contains 158 unique open reading frames, 12 of which are duplicated in the TIRs. The central region of the genome encodes genes involved in viral replication that are strongly conserved among poxviruses. Genes located towards the termini and within the TIRs are less conserved, have roles in immune evasion and host-range, and have been shown to be critical for virulence in European rabbits [16], [17].

**Figure 1:**
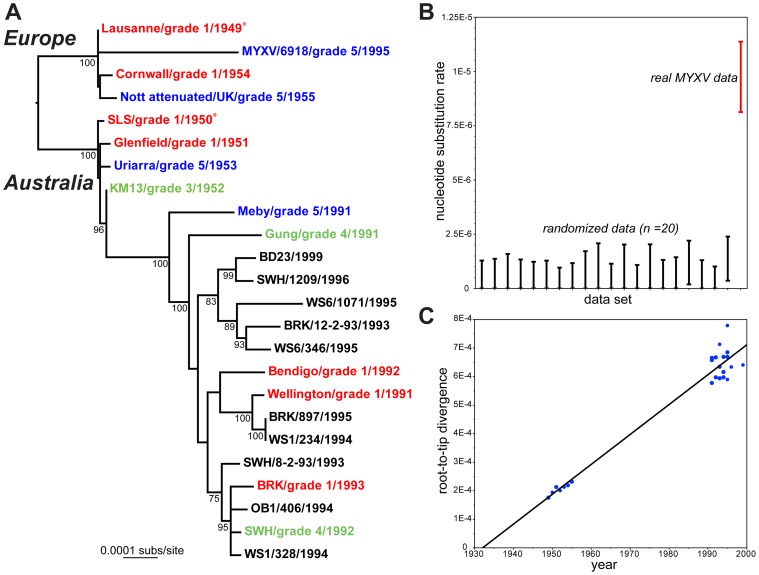
Evolutionary history of MYXV. **A:** Maximum likelihood (ML) phylogeny of 24 complete genome sequences of MYXV. Viruses are color-coded according to virulence grade (defined in Table 1); grade 1 = red, grades 3–4 = green, grade 5 = blue. The tree is rooted between the two oldest strains in the collection – Lausanne (1949) and SLS (1950) – which were used to seed the European and Australian epidemics, respectively (marked by asterisks). The same root position is obtained under the mid-point method and from the Maximum Clade Credibility (MCC) tree in BEAST [19]. All horizontal branch lengths are drawn to a scale of nucleotide substitutions per site, and bootstrap values >70% are shown. **B:** rate of the nucleotide substitution (range of 95% HPD values) for the real MYXV genomic data set (on right in red) compared to 20 sequence data sets in which the year of sampling has been randomized among the taxa. Note that the lower 95% HPD value for the randomized data tends to a zero rate. **C:** regression of root-to-tip genetic distances against year of sampling for the 24 complete genomes of MYXV and inferred from the ML phylogenetic tree. The correlation coefficient is 0.98 and the slope of the line, which is an estimator of the mean substitution rate, is 1.0×10^−5^ subs/site/year.

We obtained complete or near complete genome sequences of 22 isolates of MYXV (Table 1), with the Lu strain [16] also sequenced as a quality control. These data were combined with the published sequence of a single Spanish isolate (MYXV/6918) sampled in 1995 [18]. A maximum likelihood phylogenetic analysis of these 24 complete MYXV genomes revealed a division between those viruses released in Australia (SLS progenitor) and Europe (Lu progenitor), a strong temporal structure, such that those viruses sampled earlier in time (1940s and 1950s) fall closer to the root of the tree, and frequent changes in virulence grade over the sampling period (Figure 1a).

**Table 1:** Strains of MYXV sequenced here and their origin (in Australia unless stated otherwise).

Virus	Formal name (including year)	Geographic origin	Source	Reference	Virulence grade	Region sequenced^d^
SLS (Moses strain/strain B)	None given	Brazil	Rabbit tissue stock a(Fenner)	[3]	1	1-161777 (161763)
Glenfield	Aust/Dubbo/2-51/1	Central NSW	bCV-1 cell stock	[49]	1	15-161763 (161742)
KM13	Aust/Corowa/12-52/2	Southern NSW	Rabbit tissue stock (Fenner)	[3]	3	1-161777 (161771)
Uriarra	Aust/Uriarra/2-53/1	Canberra district	CV-1 cell stock	[49]	5	1-161777 (161768)
SWH	Aust/Southwell Hill/9-92/1	Canberra district	Wild rabbit	[22]	4	1-161777 (161797)
BRK	Aust/Brooklands/4-93	Canberra district	Wild rabbit	[22]	1	1-161777 (161701)
Bendigo	Aust/Bendigo/7-92	Central Victoria	Wild rabbit	[22]	1	1-161777 (161738)
Meby	Aust/Meby/8-91	Tasmania	Wild rabbit	[22]	5	87-161691 (161542)
Lausanne	Brazil/Campinas/1949/1	Brazil	cCommonwealth Serum Laboratories 1973	-	1	1-161777 (161778)
Cornwall	England/Cornwall/4-54/1	Cornwall UK	Rabbit tissue stock (Fenner)	[3]	1	1-161777 (161775)
Nottingham attenuated	England/Nottingham/4-55/1	Nottingham UK	Rabbit tissue stock (Fenner)	[3]	5	1-161777 (161777)
Gung	Aust/Gungahlin/1-91	Canberra district	Wild rabbit	[22]	4	151-161627 (161443)
Wellington	Aust/Wellington/1-91	Central NSW	Wild rabbit	[22]	1	29-161749 (161688)
BRK 12-2-93	Aust/Brooklands/2-93	Canberra district	Wild rabbit	[38]	ND	140-161638 (161496)
BD23	Aust/Bulloo Downs/11-99	SW Queensland	Wild rabbit	[50]	ND	285-161555 (161971)
BRK 897	Aust/Brooklands/1-95	Canberra district	Wild rabbit	[38]	ND	103-161675 (161545)
OB1 406	Aust/OB1/Hall/3-94	Canberra district	Wild rabbit	[38]	ND	87-161691 (161612)
WS1 234	Australia/Woodstock 1/3-94	Canberra district	Wild rabbit	[38]	ND	1-161777 (161754)
WS6 1071	Aust/Woodstock 6/11-95	Canberra district	Wild rabbit	[38]	ND	41-161737 (161752)
WS1 328	Aust/Woodstock 1/3-94	Canberra district	Wild rabbit	[38]	ND	156-161622 (161483)
WS6 346	Aust/Woodstock 6/3-95	Canberra district	Wild rabbit	[38]	ND	140-161638 (161430)
SWH 8-2-93	Aust/Southwell Hill/2-93	Canberra district	Wild rabbit	[38]	ND	1-161777 (161740)
SWH 1209	Aust/Southwell Hill/2-96	Canberra district	Wild rabbit	[38]	ND	33-161745 (162413)

a Virus stocks were originally obtained as freeze dried rabbit tissue from Prof. Frank Fenner, John Curtin School of Medical Research, Australian National University, Canberra, Australia.

b Virus stocks were from viruses plaque purified as described previously [49].

c Virus was from an ampoule of freeze dried rabbit tissue powder prepared by the Commonwealth Serum Laboratories for rabbit control.

d Based on the Lu sequence from Cameron et al. [16] 1-161777 as corrected by Morales et al. [18]; the actual sequence length is shown in parenthesis.

A parsimony analysis reconstructed 482 mutations across the MYXV phylogeny (mean pairwise distance among isolates = 0.05%), of which 207 were synonymous, 249 nonsynonymous, and 26 occurred in non-coding or intergenic regions. These data were also characterized by a high consistency index (0.89) indicative of limited homoplasy, and no recombination was observed. A Bayesian Markov chain Monte Carlo analysis [19] recovered a mean evolutionary rate of 9.6×10^−6^ nucleotide substitutions per site, per year (95% HPD values, 8.3–10.9×10^−6^ subs/site/year) under a strict molecular clock, with almost identical estimates obtained using a relaxed clock. Because both clock models have essentially identical likelihoods, and the coefficient of rate variation includes zero, we conclude that MYXV has evolved in a strongly clock-like manner. In addition, significant temporal structure was observed using both a phylogenetic randomization test, in which MYXV sequences with randomized times of sampling produced a distribution of substitution rates significantly different to that observed in the real MYXV data (Figure 1b), and in a regression analysis of root-to-tip genetic distances against sampling times (Figure 1c; correlation coefficient = 0.98, although this value will be inflated to some extent by phylogenetic non-independence in the data). In sum, there is measurable, and therefore relatively rapid, viral evolution over the sampling period. Accordingly, those Australian MYXV isolates sampled from the 1990s share a common ancestor that existed between 1964–1971.

### Evolution of MYXV virulence in Australia

**Figure 2:**
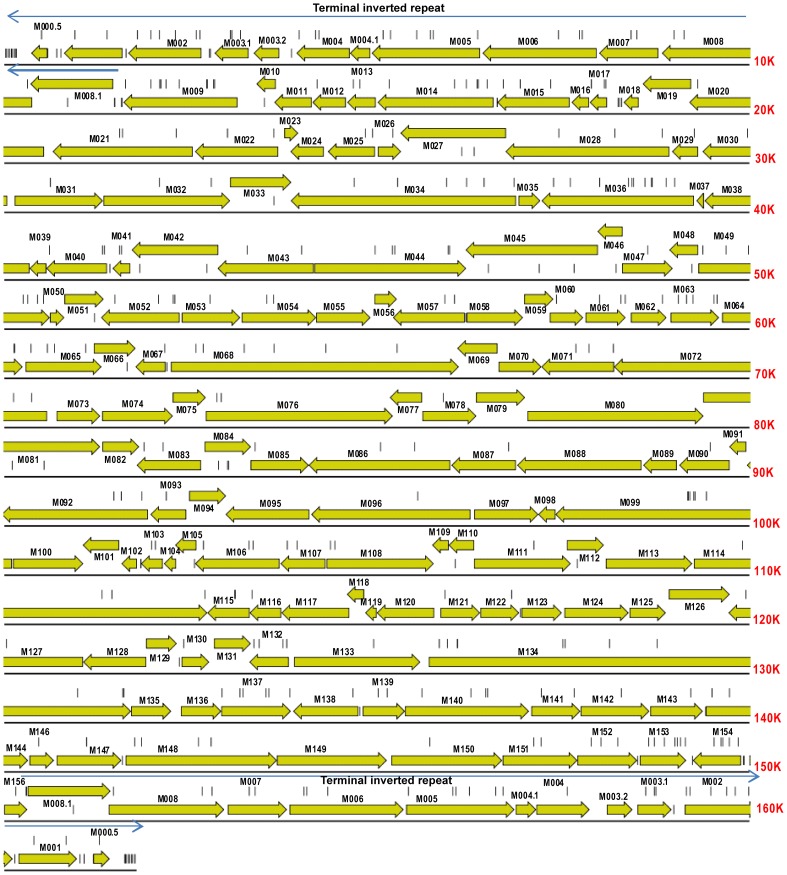
Gene map of MYXV based on the Lausanne (Lu) genome sequence (GenBank accession, NC_001132). Open reading frames and their direction of transcription are shown as arrows, and gene identities are also indicated. Light arrows above the sequence indicate the terminal inverted repeats of 11577 bp at each end of the genome. Vertical lines show the location of mutations in the Australian isolates of MYXV compared to the SLS progenitor strain that initiated the Australian epizootic of myxomatosis.

Fenner and colleagues categorised virulence on a scale from grade 1 (extreme lethality) to grade 5 (case fatality rate <50%) (Table 2). Nine of the 19 Australian MYXV isolates sequenced here have been phenotyped for virulence (Figure 1a; Table 1). These cover almost 50 years of viral evolution and the full range of virulence, including viruses that are as lethal as the progenitor SLS (grade 1) strain, as well as those with attenuated phenotypes and prolonged survival times (grade 5). Three of the phenotyped isolates were sampled during the initial epidemic; the Glenfield strain (Dubbo, Feb. 1951; grade 1), KM13 (Corowa, Dec. 1952; grade 3) and Uriarra (Feb. 1953; originally characterised as grade 4, but grade 5 in more recent testing [20]). Glenfield was more virulent than SLS when tested in resistant rabbits demonstrating that virulence can increase as well as decrease [21]. The remaining six phenotyped MYXV viruses were isolated between 1991 and 1999: Bendigo, Wellington, BRK (grade 1), SWH, Gung (grade 4) and Meby (grade 5) [22]. Among all nine phenotyped viruses there were 109 nonsynonymous mutations and 18 insertion-deletions (indels; ranging from 1–92 bp in length) in 67 open reading frames compared to the ancestral SLS sequence (Table S1). Strikingly, only two nonsynonymous (amino changes P76H in *M140R* and L204S in *M153R*) and two synonymous mutations are shared by all 19 Australian viruses compared to SLS. The genomic locations of the mutations in all 19 Australian MYXV isolates are shown in Figure 2. A total of 23 nonsynonymous, nine synonymous and one intergenic mutation fall on the branch separating the Australian viruses sampled during the 1990s from their 1950s relatives (Table S1). Interestingly, two indels are also reconstructed to fall on the branch separating the 1950s and 1990s Australian viruses; an A insert in *M009L* which disrupts this ORF (and that reverts in Bendigo), and a G insert in *M083L* that corrects a reading frame disruption in SLS and the other early Australian viruses. As MYXV will have clearly been subject to a variety of selection pressures over our sampling period, including adaptation to the European rabbit following the species jump from the tapeti, it is likely that only a subset of these mutations would be responsible for virulence evolution.

**Table 2:** Virulence grades of myxoma viruses.

Severity grade^a^	1	2	3^b^	4	5
Virulence	very high	high	moderate	low	very low
Case fatality rate	99.5%	95–99%	70–95%	50–70%	<50%
Average survival time (days)	≤13	14–16	17–28	29–50	Not defined
Prototype strain(s)	SLS; Lausanne	Not defined	KM13	Uriarra; Loiret	Neuromyxoma; Nott attenuated

a Modified from refs. 1 and 3. Virulence assays were conducted in laboratory rabbits, so that they are independent of the resistance levels of the wild rabbit as it evolved in response to the strong selection imposed by the virus. Virulence grades were assigned based on case fatality rates, average survival time and symptomatology.

b Grade 3 viruses were sometimes divided into 3A (case mortality rate of 90–95%; AST of 17–22 days) and 3B (case fatality rate of 70–90%; AST of 23–29 days) [1].

Some mutations, and particularly indels, have a clear impact on virulence. For example, the highly attenuated Uriarra strain contains a C nucleotide insertion in *M005L/R* that disrupts the reading frame. M005 is an E3 Ub ligase that manipulates cell cycle progression and inhibits cell death; as it is known to be critical for virulence, this indel is likely the main mutation responsible for the attenuation of Uriarra [23], [24]. The grade 5 Meby strain has a 73 bp deletion in the conserved region of *M153R* [22], [25] which leads to read-through of the normal stop codon and an altered C-terminal protein sequence which is no longer highly acidic. *M153R* is a key virulence gene [26], encoding an E3 Ub ligase with an N-terminal RING-CH domain responsible for downregulation of MHC-1, CD4, ALCAM/CD166 and Fas/CD95 on the membrane of infected cells [25]–[28]. Hence, this mutation is predicted to impair the capacity of MYXV to interfere with host destruction of infected cells, resulting in an attenuated phenotype.

Aside from these particular indels, the mutational events responsible for other changes in virulence are less obvious. To frame our analysis of virulence evolution, we consider the two simplest hypotheses to account for this changing MYXV phenotype in Australia. The most parsimonious explanation is that the mutations that caused attenuation in the early 1950s were maintained over the subsequent half century; hence, the phenotypic diversity in the 1990s was due to the co-circulation of virulence determinants present in the ancestral strain and attenuation mutations favoured by natural selection in the period immediately after viral release (the ‘attenuation-only’ model). A competing hypothesis is that the early MYXV strains fixed mutations that resulted in an attenuated phenotype, and that the appearance of the highly virulent grade 1 viruses in the 1990s was due to mutations which restored virulence. Under this ‘attenuation-restoration’ model patterns of phenotypic evolution over the half century following release would be due to changes in the relative frequency of strains with ancestral virulence, attenuation mutations that occurred soon after release, and strains with *de novo* mutations that restored high virulence.

Under either scenario, our genomic data can account for the observed pattern of virulence evolution only if phenotypic convergence has been achieved through multiple genetic routes. Attenuation is never associated with convergence at the genotypic level: among coding mutations, none uniquely define our single grade 3 virus, none are uniquely shared by both grade 4 viruses, and none are uniquely shared by both grade 5 viruses (Table S1). For example, the two grade 5 viruses, Meby and Uriarra, only share those nonsynonymous mutations that are fixed in all Australian strains, including those of high virulence. Similarly, no mutations are uniquely shared by the three grade 1 viruses isolated in the 1990s. Thus, if novel mutations did restore virulence to these viruses, these are specific to each. A picture of multiple genetic pathways is also observed at the genic level: none of the virulence grades can be defined by uniquely mutated genes.

Although it is clear that there are multiple routes to attenuation/virulence, mapping virulence grade on to our phylogeny using a parsimony procedure (Figure 1a) results in ambiguous state characteristics for most nodes, so that it impossible to determine the exact direction of virulence evolution. We note, however, that the ‘attenuation-only’ model cannot easily account for the phenotype of KM13, a grade 3 virus recovered late in 1952. Although KM13 differs from SLS at five amino acids, all are also found in at least one grade 1 virus. In addition, KM13 falls in a phylogenetic position that is directly ancestral to the grade 1 strains sampled in the 1990s (Figure 1a), suggesting that there was global fixation of at least some attenuating mutations as predicted by the ‘attenuation-restoration’ model.

### Evolution of MYXV in Europe

To further explore the evolution of MYXV we sequenced two British strains that have been phenotyped for virulence. Cornwall is a grade 1 virulence virus isolated in April 1954 soon after the release of MYXV in Britain [3], while Nottingham attenuated (Nott), a grade 5 virulence virus, was obtained from an infected rabbit in April 1955 [3]. We compared these viruses to both the parental Lu strain (Table S2) and to MYXV/6918, a more recent grade 5 virus from Spain.

The most likely explanation for the profound attenuation of Nott is the insertion of a TG dinucleotide in *M150R* which leads to a premature stop codon after amino acid 196. *M150R* encodes an inhibitor of NFκB, and a virus in which this gene is insertionally inactivated is highly attenuated [29], [30]. Thus, as with the M153 mutation in Meby, attenuation has apparently been achieved by the virus reducing its capacity to manipulate host immunity. This TG insertion is not present in any of the Australian viruses, and although six of the phenotyped Australian strains share a single substitution in this gene, they span the full virulence spectrum (Table S1). Similarly, single nucleotide insertions in *M135R* and *M148R* are the most likely cause of the attenuation of MYXV/6918 [18], none of which are seen in the Australian viruses. Hence, despite the parallel evolution of MYXV attenuation in Australia and Europe, different mutations in different genes were responsible for the reduction in virulence in both cases. Indeed, of the 482 mutations in the MYXV phylogeny, only two occur in parallel between the Australian and European epidemics. Both are nonsynonymous – a A37V amino acid replacement in *M003.1* and a P173S replacement in *M150* – but they are found in Australian viruses spanning the virulence spectrum and occur on the long branches separating the 1990s from the 1950s viruses.

## Discussion

Despite their importance as agents of disease, far less is known about the evolutionary dynamics of large DNA viruses than of RNA viruses, and what research has been done often relies on the assumption that the viruses in question have co-diverged with their hosts over long periods of evolutionary time [31]. Our sampling of 49 years of MYXV evolution therefore enables a unique snap-shot of the pace of viral evolution in real-time. Accordingly, our genome scale data provides strong evidence for the rapid evolution of MYXV in both Australia and Europe. The rate of nucleotide substitution we estimate here (∼1×10^−5^ subs/site/year) is one of the highest ever reported for a dsDNA virus, but similar to that documented in another poxvirus – Variola (VARV) virus – the agent of smallpox [32]. These rates are approximately three orders of magnitude higher than those estimated in the dsDNA vertebrate herpesviruses assuming virus-host co-divergence [33], and where there is a marked lack of temporal structure in the short-term [32]. It is therefore possible that poxviruses are characterized by relatively high background mutation rates, which may in part explain the clock constancy. However, direct estimates of mutation rate are only available for a limited number of dsDNA viruses, with none from the *Poxviridae* [34]. Alternatively, as MYXV was likely subject to strong selection for enhanced transmissibility and a variety of other phenotypic traits during our sampling period, it is possible that its substitution rate has been elevated by adaptive evolution. Nonsynonymous mutations were commonplace in the MYXV phylogeny, and particularly on the branch separating the 1990s Australian viruses from those sampled forty years earlier. However, their frequency of occurrence (∼55% of all mutations in coding regions) is still compatible with a predominantly neutral evolutionary process. Indeed, the small number of point mutations in individual MYXV genes precluded an analysis of gene and site-specific selection pressures.

These data are also notable in that they show that the attenuation of the grade 1 virulence of SLS in Australia was achieved by unique mutations affecting a variety of different genes. This may be a consequence of the initially wide geographic spread of MYXV which, in combination with population devastation and seasonal population bottlenecks, would have caused regionally successful virulence mutants to arise and be lost stochastically. As rabbits subsequently evolved resistance, it seems likely that a variety of virulence-restoring mutations would be locally selected among attenuated strains to maintain relative transmissibility, which was maximal for grade 4 phenotypes when measured experimentally in non-resistant laboratory rabbits [2]. Such a scenario is compatible with our MYXV phylogeny (Figure 1a) in which relatively few mutations are fixed on internal nodes of the tree, particularly during the 1950s, with more mutations located on the branches leading to individual variants and indicative of localized evolution.

More broadly, our genome scale analysis of MYXV evolution on two continents reveals that there are multiple routes to viral virulence or attenuation, of which we have likely sampled only a small number. Hence, there is convergence for phenotype but not genotype. It is likely that many of the mutational changes associated with virulence have subtle effects and are subject to complex, but as yet undefined, epistatic interactions. Such complexity could in part reflect the evolutionary arms race involving selection for resistance in rabbits and counter-selection for virulence in the virus to maintain transmissibility and competitiveness, and which led to the reversal of attenuating mutations, mutations that compensate for attenuating mutations, or those that increase virulence by novel pathways. Indeed, it is striking that both highly virulent and highly attenuated viruses appear to be successful at least under some local conditions, although the BRK and Bendigo viruses that possessed grade 1 virulence in laboratory rabbits were of grade 4 and 5 virulence, respectively, when tested in wild resistant rabbits [35], and the progenitor SLS strain was also effectively a grade 5 virus in resistant rabbits [36]. These laboratory tests were performed under cage or pen conditions and it is likely that virulence would be higher in the field due to a variety of biotic and abiotic stress factors, and which may in part explain the apparent success of attenuated viruses such as SWH or Gung. In transmission trials using viruses of virulence grades 1, 3 and 5, rabbits that died quickly following infection and those that survived infection were both poor sources of viral transmission by fleas; most transmission occurred from those rabbits that had prolonged survival times but ultimately died [37]. Stress due to nutrition, cold, and other diseases may have increased the effective virulence of a field strain of MYXV in individual rabbits, thereby enhancing the transmission of attenuated viruses and decreasing the transmission of more virulent strains. In the fragmented rabbit populations of today which are still considerably reduced from those in 1950, it may be that both local ecological conditions and virulence plasticity are critical for virus success. Clearly, multiple genetic variants of MYXV may appear in relatively small geographic areas, with the predominant viral type shifting from year to year [38].

Although *in vitro* studies of virulence determinants in viruses are commonplace, MYXV occupies a unique position in studies of virulence evolution because this evolutionary process occurred in natural populations and virulence was experimentally assayed in a controlled and relevant common garden species (laboratory rabbits of the same species as those infected in the wild). Our comparative genome sequence data suggests that the large and complex genome of MYXV provides the plasticity for multiple routes to attenuation, and multiple and complex routes back to virulence. Such genetic flexibility sits in contrast to RNA viruses such as West Nile [39], influenza A [40], and HIV-1 [41]–[43], in which it has been suggested that a limited number of mutations may control virulence. Key differences between large dsDNA viruses like MYXV and these RNA viruses are that the latter possess a limited number of genes that commonly encode multiple functions, and experience infrequent gene duplication and lateral gene transfer, all of which will limit the number of viable evolutionary pathways [44]. In light of such evolutionary complexity, it will be necessary to employ a systematic reverse genetic approach to precisely determine the contribution of individual mutations to the virulence and attenuation of this important model of disease emergence. More generally, the complex relationship between genotype and phenotype observed here suggests that it will often be difficult to predict the future course of virulence evolution in emerging pathogens from comprehensive genome sequence data.

## Materials and Methods

### Virus growth and DNA extraction

All virus isolates analyzed here, as well as their virulence, are described in Table S1. Viruses were passaged twice in RK13 cells to produce working stocks. Concentrated virions were prepared from infected RK13 cells and DNA extracted as described previously, with the exception that DNA was ethanol precipitated rather than dialysed [22].

### Sequencing and genome assembly

We obtained and assembled complete or almost complete genome sequences for 23 isolates of MYXV using the Roche GS-FLX sequencing platform. Whole genome shotgun libraries were created for each sample by fragmenting the genomic DNA using a nebulization technique. The genomic libraries were sequenced in multiple runs on the GS-FLX using the Titanium chemistry and a 16-well gasket to separate each sample on the picotiter plate (PTP). Approximately 50–60% of all the sequence reads generated corresponded to host genomic DNA so that the majority of the samples had to be sequenced twice leading to an average sequence coverage of 30× (range of 11–60X). Sequence reads were assembled into contigs using the comparative assembler AMOScmp [45] which uses a reference genome (Lu; GenBank accession NC_001132) as a guide for the assembly of related genomes, as well as with gsMapper (Roche). We also re-sequenced a Lu strain as a control of our sequencing quality. In general, the assembly produced two virus contigs per genome; one at ∼11,500 bp corresponding to the collapsed terminal inverted repeats and another, at ∼140,000 bp, corresponding to the core portion of the genome. For some genomes lighter coverage of various regions led to gaps, or suspect areas, that were closed by targeted Sanger sequencing. All high-quality reported single nucleotide polymorphisms (SNPs) were also confirmed by Sanger sequencing. SeqMan Pro (Lasergene) was used to incorporate these finishing reads into the larger assembly, followed by visual confirmation of coverage. All sequence data generated here has been submitted to GenBank and assigned accession numbers JX565562–JX565584.

### Evolutionary analysis

We first screened for recombination in the 24 complete genome sequences of MYXV (alignment length of 163,538 bp) using the RDP, GENECOV, BOOTSCAN programs available within the RDP3 package [46]. As no recombination was observed, a phylogenetic tree was then inferred using the maximum likelihood (ML) method available in the PAUP* package [47], and employing Tree Bisection and Reconnection (TBR) branch-swapping. Because of the very small number of nucleotide substitutions across the MYXV genome, such that any multiple substitution will be very limited, we utilized the HKY85 model of nucleotide substitution with default parameters. A bootstrap resampling analysis (1000 replications) was used to determine the support for key nodes. Finally, we employed a parsimony protocol to reconstruct the nucleotide changes along each branch of the ML tree, again utilizing the PAUP* package.

To infer rates of nucleotide substitution we employed the Bayesian Markov Chain Monte Carlo (MCMC) method available in the BEAST package [19]. This analysis employed both strict (i.e. constant) and relaxed (uncorrelated lognormal) molecular clocks and utilized the year of sampling of each viral isolate. We again employed the HKY85 model of nucleotide substitution as well as a Bayesian skyline coalescent prior. MCMC chains of 100 million generations (with 10% burn-in) were run multiple times until convergence was achieved for all parameters. Statistical uncertainty was reflected in values for the 95% Highest Probability Density (HPD). To determine whether there was sufficient temporal structure in the data to accurately infer evolutionary rates, we randomized sampling times 20 times across the MYXV alignment and repeated the BEAST analysis described above. In addition, we plotted root-to-tip genetic distances determined from the ML tree against year of sampling using the Path-O-Gen program (http://tree.bio.ed.ac.uk/software/pathogen/). For this analysis the ML tree is rooted between the oldest Lu and SLS strains, and which is also the deepest split in both BEAST and mid-point rooted trees.

For the BEAST analysis we used two possible dates for the SLS sequence; although the first description of this virus was published in 1911 [48], it was subjected to regular rabbit passage until 1950 when it was released into Australia. Therefore, the ‘correct’ evolutionary date for SLS falls at some point between 1911 and 1950. Although estimates of the rate of nucleotide substitution were similar under both the 1911 (95% HPD = 7.6–9.0×10^−6^ subs/site/year; strict clock) and 1950 (95% HPD = 8.3–10.9×10^−6^ subs/site/year; strict clock) dates, we based our analysis on the 1950 date as it provided a better fit to a strict molecular clock, both with respect to the coefficient of variation in BEAST (values of 0.418–1.076 and 0.00008–0.388 for the 1911 and 1950 dates, respectively) and in the root-to-tip regression (correlation coefficient = 0.92 and 0.98 for the 1911 and 1950 dates, respectively). However, the uncertainty over the correct evolutionary date for SLS does compromise estimates of when this virus shared a common ancestor with Lu.

## Supporting Information

Table S1 Sequence differences between SLS and Australian isolates of MYXV. Mutations in viruses with known phenotype are shaded.
(DOC)

Table S2 Sequence differences between Lu and the European isolates of MYXV sequenced here.
(DOC)
