# Supplementary material for: Evolutionary History and Attenuation of Myxoma Virus on Two Continents
Source: PLoS Pathog. 2012 Oct 4;8(10):e1002950. doi: 10.1371/journal.ppat.1002950 (PMC3464225; doi:10.1371/journal.ppat.1002950)
Supplement: Table S1 — Sequence differences between SLS and Australian isolates of MYXV. Mutations in viruses with known phenotype are shaded. (DOC) [file ppat.1002950.s001.doc]

**Table S1.** Sequence differences between SLS and Australian isolates of MYXV. Mutations in viruses with known phenotype are shaded.

| **SLS position** | **Sequence position** | **SLS sequence** | **Mutation** | **Virus** | **gene: change** |
| --- | --- | --- | --- | --- | --- |
| 21 | 22 | - | A | KM13 | non-coding |
| 21 | 22 | - | A | Uriarra | non-coding |
| 21 | 22 | - | A | SWH 8-2-93 | non-coding |
| 21 | 22 | - | A | WS1 234 | non-coding |
| 23 | 25 | - | A | KM13 | non-coding |
| 53 | 15 | - | G | WS6 1071 | non-coding |
| 71 | 34 | - | T | WS6 1071 | non-coding |
| 96 | 96 | A | C | BRK | non-coding |
| 113 | 11 | T | - | BRK 897 | non-coding |
| 143 | 40 | G | - | BRK 897 | non-coding |
| 148 | 110 | G | - | WS6 1071 | non-coding |
| 163 | 132 | C | T | SWH 1209 | non-coding |
| 163 | 125 | C | T | WS6 1071 | non-coding |
| 500 | 397 | G | A | BRK 897 | M00.5L: synonymous |
| 500 | 501 | G | A | WS1 234 | M00.5L: synonymous |
| 582 | 433 | G | A | Gung | intergenic |
| 704 | 554 | A | - | Gung | intergenic |
| 704 | 672 | A | - | SWH 1209 | intergenic |
| 704 | 602 | A | A | BRK 897 | intergenic |
| 704 | 706 | A | A | WS1 234 | intergenic |
| 760 | 610 | C | T | Gung | intergenic |
| 947 | 949 | C | T | WS1 234 | M001L: S213N |
| 947 | 920 | C | T | Wellington | M001L: S213N |
| 947 | 845 | C | T | BRK 897 | M001L: S213N |
| 1379 | 1241 | C | T | BRK 12-2-93 | M001L: G69D |
| 1634 | 1608 | - | T | Wellington | intergenic |
| 1787 | 1649 | C | T | BRK 12-2-93 | M002L: synonymous |
| 1968 | 1883 | G | A | OB1 406 | M002L: A226V |
| 1968 | 1970 | G | A | WS1 234 | M002L: A226V |
| 1968 | 1930 | G | A | WS6 1071 | M002L: A226V |
| 1968 | 1814 | C | A | WS1 328 | M002L: A226V |
| 1968 | 1830 | G | A | WS6 346 | M002L: A226V |
| 1968 | 1685 | G | A | BD23 | M002L: A226V |
| 1968 | 1936 | G | A | SWH 1209 | M002L: A226V |
| 1968 | 1969 | G | A | SWH 8-2-93 | M002L: A226V |
| 1968 | 1968 | G | A | BRK | M002L: A226V |
| 1968 | 1968 | G | A | SWH | M002L: A226V |
| 1968 | 1883 | G | A | Meby | M002L: A226V |
| 1968 | 1942 | G | A | Wellington | M002L: A226V |
| 1968 | 1968 | G | A | Bendigo | M002L: A226V |
| 1968 | 1818 | G | A | Gung | M002L: A226V |
| 1968 | 1866 | G | A | BRK 897 | M002L: A226V |
| 1968 | 1830 | G | A | BRK 12-2-93 | M002L: A226V |
| 2083 | 1933 | T | C | Gung | M002L: T118A |
| 2295 | 2295 | T | C | Bendigo | M002L: Q117R |
| 2295 | 2145 | T | C | Gung | M002L: Q117R |
| 2576 | 2426 | T | C | Gung | M002L: synonymous |
| 2576 | 2438 | T | C | BRK 12-2-93 | M002L: synonymous |
| 2576 | 2550 | T | C | Wellington | M002L: synonymous |
| 2576 | 2293 | T | C | BD23 | M002L: synonymous |
| 2576 | 2474 | T | C | BRK 897 | M002L: synonymous |
| 2576 | 2491 | T | C | OB1 406 | M002L: synonymous |
| 2576 | 2578 | T | C | WS1 234 | M002L: synonymous |
| 2576 | 2538 | T | C | WS6 1071 | M002L: synonymous |
| 2576 | 2422 | T | C | WS1 328 | M002L: synonymous |
| 2576 | 2438 | T | C | WS6 346 | M002L: synonymous |
| 2576 | 2577 | T | C | SWH 8-2-93 | M002L: synonymous |
| 2576 | 2544 | T | C | SWH 1209 | M002L: synonymous |
| 2576 | 2562 | T | C | Glenfield | M002L: synonymous |
| 2576 | 2576 | T | C | BRK | M002L: synonymous |
| 2576 | 2576 | T | C | SWH | M002L: synonymous |
| 2576 | 2491 | T | C | Meby | M002L: synonymous |
| 2576 | 2576 | T | C | Bendigo | M002L: synonymous |
| 2576 | 2578 | T | C | KM13 | M002L: synonymous |
| 2576 | 2577 | T | C | Uriarra | M002L: synonymous |
| 2624 | 2539 | C | T | OB1 406 | M002L: synonymous |
| 2624 | 2470 | T | C | WS1 328 | M002L: synonymous |
| 2624 | 2624 | T | C | BRK | M002L: synonymous |
| 2624 | 2624 | T | C | SWH | M002L: synonymous |
| 2772 | 2633 |  | 12 nt del | WS6 346 | intergenic |
| 2798 | 2799 |  | 27 nt del | WS1 234 | intergenic |
| 2798 | 2797 |  | 27 nt del | Bendigo | intergenic |
| 2798 | 2771 |  | 27 nt del | Wellington | intergenic |
| 2798 | 2647 |  | 27 nt del | Gung | intergenic |
| 2798 | 2695 |  | 27 nt del | BRK 897 | intergenic |
| 2798 | 2798 |  | 27 nt del | SWH 8-2-93 | intergenic |
| 2798 | 2647 |  | 27 nt del | WS6 346 | intergenic |
| 3031 | 2993 | G | A | WS6 1071 | M003.1L: H83Y |
| 3052 | 3027 | G | C | WS1 234 | M003.1L: L76V |
| 3052 | 3026 | G | C | SWH 8-2-93 | M003.1L: L76V |
| 3052 | 3025 | G | C | Bendigo | M003.1L: L76V |
| 3052 | 2923 | G | C | BRK 897 | M003.1L: L76V |
| 3052 | 2999 | G | T | Wellington | M003.1L: L76V |
| 3168 | 3083 | G | A | OB1 406 | M003.1L: A37V |
| 3168 | 3143 | G | A | WS1 234 | M003.1L: A37V |
| 3168 | 3130 | G | A | WS6 1071 | M003.1L: A37V |
| 3168 | 3014 | G | A | WS1 328 | M003.1L: A37V |
| 3168 | 2991 | G | A | WS6 346 | M003.1L: A37V |
| 3168 | 3115 | G | A | Wellington | M003.1L: A37V |
| 3168 | 3142 | G | A | SWH 8-2-93 | M003.1L: A37V |
| 3168 | 3168 | G | A | BRK | M003.1L: A37V |
| 3168 | 3168 | G | A | SWH | M003.1L: A37V |
| 3168 | 3083 | G | A | Meby | M003.1L: A37V |
| 3168 | 3136 | G | A | SWH 1209 | M003.1L: A37V |
| 3168 | 2885 | G | A | BD23 | M003.1L: A37V |
| 3168 | 2991 | G | A | Gung | M003.1L: A37V |
| 3168 | 3039 | G | A | BRK 897 | M003.1L: A37V |
| 3168 | 3030 | G | A | BRK 12-2-93 | M003.1L: A37V |
| 3168 | 3141 | G | A | Bendigo | M003.1L: A37V |
| 3854 | 3821 | A | - | SWH 1209 | intergenic |
| 3956 | 3956 | G | A | SWH | M004L: P227S |
| 4176 | 4138 | C | T | WS6 1071 | M004L: synonymous |
| 4176 | 3999 | C | T | Gung | M004L: synonymous |
| 4221 | 4136 | G | C | Meby | M004L: N138K |
| 5082 | 4997 | G | A | OB1 406 | M005L: R434W |
| 5082 | 5057 | G | A | WS1 234 | M005L: R434W |
| 5082 | 5044 | G | A | WS6 1071 | M005L: R434W |
| 5082 | 4928 | G | A | WS1 328 | M005L: R434W |
| 5082 | 4905 | G | A | WS6 346 | M005L: R434W |
| 5082 | 5082 | G | A | BRK | M005L: R434W |
| 5082 | 5029 | G | A | Wellington | M005L: R434W |
| 5082 | 5082 | G | A | SWH | M005L: R434W |
| 5082 | 4997 | G | A | Meby | M005L: R434W |
| 5082 | 5055 | G | A | Bendigo | M005L: R434W |
| 5082 | 5056 | G | A | SWH 8-2-93 | M005L: R434W |
| 5082 | 4905 | G | A | Gung | M005L: R434W |
| 5082 | 4953 | G | A | BRK 897 | M005L: R434W |
| 5082 | 4944 | G | A | BRK 12-2-93 | M005L: R434W |
| 5082 | 4799 | G | A | BD23 | M005L: R434W |
| 5082 | 5049 | G | A | SWH 1209 | M005L: R434W |
| 5082 | 5044 | G | A | WS6 1071 | M005L: synonymous |
| 5533 | 5357 | - | C | WS6 346 | M005L: frameshift |
| 5716 | 5562 | T | C | WS1 328 | M005L: synonymous |
| 5756 | 5671 | G | T | OB1 406 | M005L: S209Y |
| 5756 | 5731 | G | T | WS1 234 | M005L: S209Y |
| 5756 | 5718 | G | T | WS6 1071 | M005L: S209Y |
| 5756 | 5602 | G | T | WS1 328 | M005L: S209Y |
| 5756 | 5579 | G | T | Gung | M005L: S209Y |
| 5756 | 5580 | G | T | WS6 346 | M005L: S209Y |
| 5756 | 5473 | G | T | BD23 | M005L: S209Y |
| 5756 | 5723 | G | T | SWH 1209 | M005L: S209Y |
| 5756 | 5730 | G | T | SWH 8-2-93 | M005L: S209Y |
| 5756 | 5756 | G | T | BRK | M005L: S209Y |
| 5756 | 5756 | G | T | SWH | M005L: S209Y |
| 5756 | 5671 | G | T | Meby | M005L: S209Y |
| 5756 | 5729 | G | T | Bendigo | M005L: S209Y |
| 5756 | 5703 | G | T | Wellington | M005L: S209Y |
| 5756 | 5627 | G | T | BRK 897 | M005L: S209Y |
| 5756 | 6618 | G | T | BRK 12-2-93 | M005L: S209Y |
| 6352 | 6354 | - | G | Uriarra | M005: frameshift |
| 7436 | 7259 | T | C | Gung | M006L: D171G |
| 7717 | 7684 | T | C | SWH 1209 | M006L: I77M |
| 7755 | 7617 | T | G | BRK 12-2-93 | M006L: T65P |
| 8410 | 8372 | G | A | WS6 1071 | M007L: synonymous |
| 8489 | 8451 | G | A | WS6 1071 | M007L: A96V |
| 8988 | 8903 | G | A | OB1 406 | M008L: synonymous |
| 8988 | 8834 | G | A | WS1 328 | M008L: synonymous |
| 8988 | 8705 | G | A | BD23 | M008L: synonymous |
| 8988 | 8955 | G | A | SWH 1209 | M008L: synonymous |
| 8988 | 8962 | G | A | SWH 8-2-93 | M008L: synonymous |
| 8988 | 8988 | G | A | BRK | M008L: synonymous |
| 8988 | 8988 | G | A | SWH | M008L: synonymous |
| 8988 | 8961 | G | A | Bendigo | M008L: synonymous |
| 9121 | 8945 | G | A | WS6 346 | M008L: A418V |
| 10311 | 10226 | G | A | Meby | M008L: synonymous |
| 10849 | 10673 | C | T | WS6 346 | M008.1: synonymous |
| 11477 | 11300 | A | - | WS6 346 | intergenic |
| 11484 | 11346 | A | G | BRK 12-2-93 | intergenic |
| 11484 | 11431 | A | G | Wellington | intergenic |
| 11484 | 11355 | A | G | BRK 897 | intergenic |
| 11484 | 11459 | A | G | WS1 234 | intergenic |
| 11484 | 11446 | A | G | WS6 1071 | intergenic |
| 11484 | 11307 | A | G | WS6 346 | intergenic |
| 11484 | 11201 | A | G | BD23 | intergenic |
| 11484 | 11451 | A | G | SWH 1209 | intergenic |
| 11576 | 11544 |  |  | SWH 1209 | 11544-13178: 1635 nt insertion |
| 11576 | 11294 |  |  | BD23 | 11294-12928: 1635 nt insertion |
| 11577 | 13178 |  |  | SWH 1209 | M009: deletion of 3’ 923 nucleotides |
| 11577 | 12928 |  |  | BD23 | M009: deletion of 3’ 923 nucleotides |
| 11626 | 11599 | A | - | SWH 8-2-93 | M009L: frameshift |
| 11966 | 11828 | C | T | BRK 12-2-93 | M009: synonymous |
| 12086 | 12001 | C | T | Meby | M009L: TGG>TGA stop codon |
| 12102 | 12017 | G | A | Meby | M009L: P343L |
| 12170 | 12171 | - | T | BRK | M009L: frameshift |
| 12170 | 12172 | - | A | BRK | M009L: frameshift |
| 12348 | 12171 | G | A | Gung | M009L: A261V |
| 12348 | 12210 | G | A | BRK 12-2-93 | M009L: A261V |
| 12348 | 12295 | G | A | Wellington | M009L: A261V |
| 12348 | 12219 | G | A | BRK 897 | M009L: A261V |
| 12348 | 12263 | G | A | OB1 406 | M009L: A261V |
| 12348 | 12323 | G | A | WS1 234 | M009L: A261V |
| 12348 | 12310 | G | A | WS6 1071 | M009L: A261V |
| 12348 | 12194 | G | A | WS1 328 | M009L: A261V |
| 12348 | 12171 | G | A | WS6 346 | M009L: A261V |
| 12348 | 12321 | G | A | SWH 8-2-93 | M009L: A261V |
| 12348 | 12350 | G | A | BRK | M009L: A261V |
| 12348 | 12348 | G | A | SWH | M009L: A261V |
| 12348 | 12321 | G | A | Bendigo | M009L: A261V |
| 12715 | 12539 | - | A | Gung | M009L: frameshift |
| 12715 | 12718 | - | A | BRK | M009L: frameshift |
| 12715 | 12578 | - | A | BRK 12-2-93 | M009L: frameshift |
| 12715 | 12663 | - | A | Wellington | M009L: frameshift |
| 12715 | 13122 | - | A | BD23 | M009L: frameshift |
| 12715 | 12587 | - | A | BRK 897 | M009L: frameshift |
| 12715 | 12631 | - | A | OB1 406 | M009L: frameshift |
| 12715 | 12691 | - | A | WS1 234 | M009L: frameshift |
| 12715 | 12678 | - | A | WS6 1071 | M009L: frameshift |
| 12715 | 12562 | - | A | WS1 328 | M009L: frameshift |
| 12715 | 12539 | - | A | WS6 346 | M009L: frameshift |
| 12715 | 12689 | - | A | SWH 8-2-93 | M009L: frameshift |
| 12715 | 13372 | - | A | SWH 1209 | M009L: frameshift |
| 12715 | 12722 | - | A | SWH | M009L: frameshift |
| 12715 | 12620 | - | A | Meby | M009L: frameshift |
| 12724 | 12640 | C | T | Meby | M009L: A140T |
| 12809 | 12771 | A | - | WS6 1071 | M009L: frameshift |
| 12823 | 12785 | C | T | WS6 1071 | M009L: E103K |
| 13490 | 13337 | A | G | WS1 328 | M010L: synonymous |
| 14198 | 14146 | C | T | Wellington | M012L: S129N |
| 14198 | 14070 | C | T | BRK 897 | M012L: S129N |
| 14198 | 14174 | C | T | WS1 234 | M012L: S129N |
| 14684 | 14600 | T | C | Meby | M13L: synonymous |
| 14772 | 14635 | T | C | BRK 12-2-93 | M13L: synonymous |
| 15164 | 15151 | - | G | Glenfield | M014L: frameshift |
| 15164 | 15167 | - | G | KM13 | M014L: frameshift |
| 15164 | 15167 | - | G | Uriarra | M014L: frameshift |
| 16042 | 15866 | C | T | Gung | M014L: V175I |
| 16042 | 15905 | C | T | BRK 12-2-93 | M014L: V175I |
| 16042 | 15990 | C | T | Wellington | M014L: V175I |
| 16042 | 16449 | C | T | BD23 | M014L: V175I |
| 16042 | 15914 | C | T | BRK 897 | M014L: V175I |
| 16042 | 15958 | C | T | OB1 406 | M014L: V175I |
| 16042 | 16018 | C | T | WS1 234 | M014L: V175I |
| 16042 | 16004 | C | T | WS6 1071 | M014L: V175I |
| 16042 | 15889 | C | T | WS1 328 | M014L: V175I |
| 16042 | 15866 | C | T | WS6 346 | M014L: V175I |
| 16042 | 16016 | C | T | SWH 8-2-93 | M014L: V175I |
| 16042 | 16699 | C | T | SWH 1209 | M014L: V175I |
| 16042 | 16045 | C | T | BRK | M014L: V175I |
| 16042 | 16043 | C | T | SWH | M014L: V175I |
| 16042 | 15958 | C | T | Meby | M014L: V175I |
| 16042 | 16015 | C | T | Bendigo | M014L: V175I |
| 16201 | 16025 | C | A | Gung | M014L: G122W |
| 16201 | 16064 | C | A | BRK 12-2-93 | M014L: G122W |
| 16201 | 16149 | C | A | Wellington | M014L: G122W |
| 16201 | 16608 | C | A | BD23 | M014L: G122W |
| 16201 | 16073 | C | A | BRK 897 | M014L: G122W |
| 16201 | 16117 | C | A | OB1 406 | M014L: G122W |
| 16201 | 16177 | C | A | WS1 234 | M014L: G122W |
| 16201 | 16163 | C | A | WS6 1071 | M014L: G122W |
| 16201 | 16048 | C | A | WS1 328 | M014L: G122W |
| 16201 | 16025 | C | A | WS6 346 | M014L: G122W |
| 16201 | 16175 | C | A | SWH 8-2-93 | M014L: G122W |
| 16201 | 16858 | C | A | SWH 1209 | M014L: G122W |
| 16201 | 16204 | C | A | BRK | M014L: G122W |
| 16201 | 16202 | C | A | SWH | M014L: G122W |
| 16201 | 16117 | C | A | Meby | M014L: G122W |
| 16201 | 16174 | C | A | Bendigo | M014L: G122W |
| 16346 | 16170 | G | A | WS6 346 | M014L: synonymous |
| 16361 | 16362 | G | A | SWH | M014L: synonymous |
| 16478 | 16302 | G | A | Gung | M014L: synonymous |
| 16478 | 16426 | G | A | Wellington | M014L: synonymous |
| 16478 | 16885 | G | A | BD23 | M014L: synonymous |
| 16478 | 16350 | G | A | BRK 897 | M014L: synonymous |
| 16478 | 16394 | G | A | OB1 406 | M014L: synonymous |
| 16478 | 16454 | G | A | WS1 234 | M014L: synonymous |
| 16478 | 16440 | G | A | WS6 1071 | M014L: synonymous |
| 16478 | 16325 | G | A | WS1 328 | M014L: synonymous |
| 16478 | 16452 | G | A | SWH 8-2-93 | M014L: synonymous |
| 16478 | 17135 | G | A | SWH 1209 | M014L: synonymous |
| 16478 | 16481 | G | A | BRK | M014L: synonymous |
| 16478 | 16479 | G | A | SWH | M014L: synonymous |
| 16478 | 16451 | G | A | Bendigo | M014L: synonymous |
| 16614 | 17020 | A | - | BD23 | intergenic |
| 16615 | 17020 | A | - | BD23 | intergenic |
| 16615 | 16477 | A | - | BRK 12-2-93 | intergenic |
| 16615 | 16530 | A | - | OB1 406 | intergenic |
| 16615 | 16461 | A | - | WS1 328 | intergenic |
| 16615 | 16438 | A | - | WS6 346 | intergenic |
| 16615 | 16588 | A | - | SWH 8-2-93 | intergenic |
| 16615 | 17271 | A | - | SWH 1209 | intergenic |
| 16615 | 16617 | A | - | Uriarra | intergenic |
| 16615 | 16615 | A | - | SWH | intergenic |
| 16615 | 16587 | A | - | Bendigo | intergenic |
| 16615 | 16440 | - | A | Gung | intergenic |
| 16695 | 16557 | T | C | BRK 12-2-93 | M015L: synonymous |
| 16923 | 16748 | T | C | Gung | M015L: synonymous |
| 16923 | 16785 | T | C | BRK 12-2-93 | M015L: synonymous |
| 16923 | 16871 | T | C | Wellington | M015L: synonymous |
| 16923 | 17328 | T | C | BD23 | M015L: synonymous |
| 16923 | 16795 | T | C | BRK 897 | M015L: synonymous |
| 16923 | 16838 | T | C | OB1 406 | M015L: synonymous |
| 16923 | 16899 | T | C | WS1 234 | M015L: synonymous |
| 16923 | 16885 | T | C | WS6 1071 | M015L: synonymous |
| 16923 | 16769 | T | C | WS1 328 | M015L: synonymous |
| 16923 | 16746 | T | C | WS6 346 | M015L: synonymous |
| 16923 | 16896 | T | C | SWH 8-2-93 | M015L: synonymous |
| 16923 | 17579 | T | C | SWH 1209 | M015L: synonymous |
| 16923 | 16826 | T | C | BRK | M015L: synonymous |
| 16923 | 16923 | T | C | SWH | M015L: synonymous |
| 16923 | 16839 | T | C | Meby | M015L: synonymous |
| 16923 | 16895 | T | C | Bendigo | M015L: synonymous |
| 17332 | 17157 | A | G | Gung | M015L: V85A |
| 17332 | 17194 | A | G | BRK 12-2-93 | M015L: V85A |
| 17332 | 17280 | A | G | Wellington | M015L: V85A |
| 17332 | 17737 | A | G | BD23 | M015L: V85A |
| 17332 | 17204 | A | G | BRK 897 | M015L: V85A |
| 17332 | 17247 | A | G | OB1 406 | M015L: V85A |
| 17332 | 17308 | A | G | WS1 234 | M015L: V85A |
| 17332 | 17294 | A | G | WS6 1071 | M015L: V85A |
| 17332 | 17178 | A | G | WS1 328 | M015L: V85A |
| 17332 | 17155 | A | G | WS6 346 | M015L: V85A |
| 17332 | 17305 | A | G | SWH 8-2-93 | M015L: V85A |
| 17332 | 17988 | A | G | SWH 1209 | M015L: V85A |
| 17332 | 17334 | A | G | BRK | M015L: V85A |
| 17332 | 17332 | A | G | SWH | M015L: V85A |
| 17332 | 17248 | A | G | Meby | M015L: V85A |
| 17332 | 17304 | A | G | Bendigo | M015L: V85A |
| 17877 | 17702 | C | T | Gung | M017L: E71K |
| 17877 | 17739 | C | T | BRK 12-2-93 | M017L: E71K |
| 17877 | 17825 | C | T | Wellington | M017L: E71K |
| 17877 | 18282 | C | T | BD23 | M017L: E71K |
| 17877 | 17749 | C | T | BRK 897 | M017L: E71K |
| 17877 | 17792 | C | T | OB1 406 | M017L: E71K |
| 17877 | 17853 | C | T | WS1 234 | M017L: E71K |
| 17877 | 17839 | C | T | WS6 1071 | M017L: E71K |
| 17877 | 17723 | C | T | WS1 328 | M017L: E71K |
| 17877 | 17700 | C | T | WS6 346 | M017L: E71K |
| 17877 | 17850 | C | T | SWH 8-2-93 | M017L: E71K |
| 17877 | 18533 | C | T | SWH 1209 | M017L: E71K |
| 17877 | 17879 | C | T | BRK | M017L: E71K |
| 17877 | 17877 | C | T | SWH | M017L: E71K |
| 17877 | 17793 | C | T | Meby | M017L: E71K |
| 17877 | 17849 | C | T | Bendigo | M017L: E71K |
| 17878 | 17840 | G | C | WS6 1071 | M017L: synonymous |
| 18052 | 18457 | C | T | BD23 | M017L: M12I |
| 18073 | 17898 | G | A | Gung | M017L: synonymous |
| 18236 | 18239-18254 |  | 16 nt insert | BRK | intergenic |
|  | 18209-18224 |  | 16 nt insert | Bendigo | intergenic |
|  | 18185-18200 |  | 16 nt insert | Wellington | intergenic |
|  | 18062-18085 |  | 24 nt insert | Gung | intergenic |
|  | 18237-18252 |  | 16 nt insert | SWH | intergenic |
|  | 18153-18168 |  | 16 nt insert | Meby | intergenic |
|  | 18099-18106 |  | 8 nt insert | BRK 12-2-93 | intergenic |
|  | 18109-18124 |  | 16 nt insert | BRK 897 | intergenic |
|  | 18152-18159 |  | 8 nt insert | OB1 406 | intergenic |
|  | 18893-18908 |  | 16 nt insert | SWH 1209 | intergenic |
|  | 18642-18657 |  | 16 nt insert | BD23 | intergenic |
|  | 18210-18225 |  | 16 nt insert | SWH 8-2-93 | intergenic |
|  | 18213-18228 |  | 16 nt insert | WS1 234 | intergenic |
|  | 18083-18098 |  | 16 nt insert | WS1 328 | intergenic |
|  | 18199-18262 |  | 64 nt insert | WS6 1071 | intergenic |
|  | 18060-18075 |  | 16 nt insert | WS6 346 | intergenic |
| 18250 | 18269-18276 |  | 8 nt insert | BRK | intergenic |
|  | 18239-18246 |  | 8 nt insert | Bendigo | intergenic |
|  | 18215-18230 |  | 16 nt insert | Wellington | intergenic |
|  | 18100-18107 |  | 8 nt insert | Gung | intergenic |
|  | 18267-18274 |  | 8 nt insert | SWH | intergenic |
|  | 18139-18154 |  | 16 nt insert | BRK 897 | intergenic |
|  | 18174-18181 |  | 8 nt insert | OB1 406 | intergenic |
|  | 18923-18930 |  | 8 nt insert | SWH 1209 | intergenic |
|  | 18672-18679 |  | 8 nt insert | BD23 | intergenic |
|  | 18240-18247 |  | 8 nt insert | SWH 8-2-93 | intergenic |
|  | 18243-18258 |  | 16 nt insert | WS1 234 | intergenic |
|  | 18113-18120 |  | 8 nt insert | WS1 328 | intergenic |
|  | 19090-18105 |  | 16 nt insert | WS6 346 | intergenic |
| 18277 | 18134 | T | C | Gung | intergenic |
| 18277 | 18147 | T | C | BRK 12-2-93 | intergenic |
| 18277 | 18257 | T | C | Wellington | intergenic |
| 18277 | 18706 | T | C | BD23 | intergenic |
| 18277 | 18181 | T | C | BRK 897 | intergenic |
| 18277 | 18208 | T | C | OB1 406 | intergenic |
| 18277 | 18285 | T | C | WS1 234 | intergenic |
| 18277 | 18303 | T | C | WS6 1071 | intergenic |
| 18277 | 18147 | T | C | WS1 328 | intergenic |
| 18277 | 18132 | T | C | WS6 346 | intergenic |
| 18277 | 18274 | T | C | SWH 8-2-93 | intergenic |
| 18277 | 18957 | T | C | SWH 1209 | intergenic |
| 18277 | 18303 | T | C | BRK | intergenic |
| 18277 | 18301 | T | C | SWH | intergenic |
| 18277 | 18209 | T | C | Meby | intergenic |
| 18277 | 18273 | T | C | Bendigo | intergenic |
| 19296 | 19725 | G | A | BD23 | M020L: synonymous |
| 21539 | 21519 | G | A | Wellington | M021L: R328C |
| 21539 | 21443 | G | A | BRK 897 | M021L: R328C |
| 21539 | 21470 | G | A | OB1 406 | M021L: R328C |
| 21539 | 21547 | G | A | WS1 234 | M021L: R328C |
| 21539 | 21409 | G | A | WS1 328 | M021L: R328C |
| 21539 | 21536 | G | A | SWH 8-2-93 | M021L: R328C |
| 21539 | 21565 | G | A | BRK | M021L: R328C |
| 21539 | 21563 | G | A | SWH | M021L: R328C |
| 21539 | 21535 | G | A | Bendigo | M021L: R328C |
| 21578 | 21435 | C | T | Gung | M021L: D315N |
| 21578 | 21558 | C | T | Wellington | M021L: D315N |
| 21578 | 21482 | C | T | BRK 897 | M021L: D315N |
| 21578 | 21509 | C | T | OB1 406 | M021L: D315N |
| 21578 | 21586 | C | T | WS1 234 | M021L: D315N |
| 21578 | 21604 | C | T | WS6 1071 | M021L: D315N |
| 21578 | 21448 | C | T | WS1 328 | M021L: D315N |
| 21578 | 21575 | C | T | SWH 8-2-93 | M021L: D315N |
| 21578 | 21604 | C | T | BRK | M021L: D315N |
| 21578 | 21602 | C | T | SWH | M021L: D315N |
| 21578 | 21574 | C | T | Bendigo | M021L: D315N |
| 22299 | 22230 | G | A | OB1 406 | M021L: synonymous |
| 22299 | 22169 | G | A | WS1 328 | M021L: synonymous |
| 22299 | 22296 | G | A | SWH 8-2-93 | M021L: synonymous |
| 22299 | 22325 | G | A | BRK | M021L: synonymous |
| 22299 | 22323 | G | A | SWH | M021L: synonymous |
| 22299 | 22295 | G | A | Bendigo | M021L: synonymous |
| 22987 | 22984 | C | T | SWH 8-2-93 | M021L: synonymous |
| 23608 | 23465 | G | A | Gung | M022L: synonymous |
| 23608 | 23478 | G | A | BRK 12-2-93 | M022L: synonymous |
| 23608 | 23588 | G | A | Wellington | M022L: synonymous |
| 23608 | 24037 | G | A | BD23 | M022L: synonymous |
| 23608 | 23512 | G | A | BRK 897 | M022L: synonymous |
| 23608 | 23539 | G | A | OB1 406 | M022L: synonymous |
| 23608 | 23616 | G | A | WS1 234 | M022L: synonymous |
| 23608 | 23634 | G | A | WS6 1071 | M022L: synonymous |
| 23608 | 23478 | G | A | WS1 328 | M022L: synonymous |
| 23608 | 23463 | G | A | WS6 346 | M022L: synonymous |
| 23608 | 23605 | G | A | SWH 8-2-93 | M022L: synonymous |
| 23608 | 24288 | G | A | SWH 1209 | M022L: synonymous |
| 23608 | 23634 | G | A | BRK | M022L: synonymous |
| 23608 | 23632 | G | A | SWH | M022L: synonymous |
| 23608 | 23540 | G | A | Meby | M022L: synonymous |
| 23608 | 23604 | G | A | Bendigo | M022L: synonymous |
| 24837 | 24707 | G | A | BRK 12-2-93 | M025L: synonymous |
| 24837 | 25266 | G | A | BD23 | M025L: synonymous |
| 24837 | 24692 | G | A | WS6 346 | M025L: synonymous |
| 24837 | 25517 | G | A | SWH 1209 | M025L: synonymous |
| 24933 | 24790 | C | T | Gung | M025L:M11I |
| 24933 | 24803 | C | T | BRK 12-2-93 | M025L:M11I |
| 24933 | 24913 | C | T | Wellington | M025L:M11I |
| 24933 | 25362 | C | T | BD23 | M025L:M11I |
| 24933 | 24837 | C | T | BRK 897 | M025L:M11I |
| 24933 | 24864 | C | T | OB1 406 | M025L:M11I |
| 24933 | 24941 | C | T | WS1 234 | M025L:M11I |
| 24933 | 24959 | C | T | WS6 1071 | M025L:M11I |
| 24933 | 24803 | C | T | WS1 328 | M025L:M11I |
| 24933 | 24788 | C | T | WS6 346 | M025L:M11I |
| 24933 | 24930 | C | T | SWH 8-2-93 | M025L:M11I |
| 24933 | 25613 | C | T | SWH 1209 | M025L:M11I |
| 24933 | 24959 | C | T | BRK | M025L:M11I |
| 24933 | 24957 | C | T | SWH | M025L:M11I |
| 24933 | 24929 | C | T | Bendigo | M025L:M11I |
| 25206 | 25063 | C | T | Gung | M026R: synonymous |
| 25206 | 25076 | C | T | BRK 12-2-93 | M026R: synonymous |
| 25206 | 25635 | C | T | BD23 | M026R: synonymous |
| 25206 | 25232 | C | T | WS6 1071 | M026R: synonymous |
| 25206 | 25061 | C | T | WS6 346 | M026R: synonymous |
| 25206 | 25886 | C | T | SWH 1209 | M026R: synonymous |
| 26130 | 26062 | C | T | Meby | M027L: synonymous |
| 26297 | 26154 | C | T | Gung | M027L: D143N |
| 26297 | 26323 | C | T | WS6 1071 | M027L: D143N |
| 26297 | 26293 | C | T | Bendigo | M027L: D143N |
| 27433 | 27862 | G | A | BD23 | M028L: R495C |
| 28185 | 28092 | G | A | Gung | M028L: S244L |
| 28185 | 28165 | G | A | Wellington | M028L: S244L |
| 28185 | 28614 | G | A | BD23 | M028L: S244L |
| 28185 | 28089 | G | A | BRK 897 | M028L: S244L |
| 28185 | 28116 | G | A | OB1 406 | M028L: S244L |
| 28185 | 28193 | G | A | WS1 234 | M028L: S244L |
| 28185 | 28211 | G | A | WS6 1071 | M028L: S244L |
| 28185 | 28055 | G | A | WS1 328 | M028L: S244L |
| 28185 | 28182 | G | A | SWH 8-2-93 | M028L: S244L |
| 28185 | 28865 | G | A | SWH 1209 | M028L: S244L |
| 28185 | 28211 | G | A | BRK | M028L: S244L |
| 28185 | 28209 | G | A | SWH | M028L: S244L |
| 28185 | 28181 | G | A | Bendigo | M028L: S244L |
| 28819 | 28751 | G | A | Meby | M028L: R33C |
| 29252 | 29122 | G | A | BRK 12-2-93 | M029L: A17V |
| 29252 | 29681 | G | A | BD23 | M029L: A17V |
| 29252 | 29183 | G | A | OB1 406 | M029L: A17V |
| 29252 | 29122 | G | A | WS1 328 | M029L: A17V |
| 29252 | 29107 | G | A | WS6 346 | M029L: A17V |
| 29252 | 29249 | G | A | SWH 8-2-93 | M029L: A17V |
| 29252 | 29932 | G | A | SWH 1209 | M029L: A17V |
| 29252 | 29278 | G | A | BRK | M029L: A17V |
| 29252 | 29276 | G | A | SWH | M029L: A17V |
| 29966 | 29821 | C | T | WS6 346 | M030L: synonymous |
| 30611 | 30607 | C | T | Bendigo | M031R: synonymous |
| 32075 | 31945 | C | T | WS1 328 | M032R: synonymous |
| 32769 | 32749 | A | G | Wellington | M032R: I481V |
| 32769 | 32765 | A | G | Bendigo | M032R: I481V |
| 33610 | 33636 | C | T | WS6 1071 | M033R: synonymous |
| 34687 | 35116 | G | A | BD23 | M034L: synonymous |
| 35269 | 35256 | T | C | Glenfield | M034L: synonymous |
| 35926 | 35830 | G | A | BRK 897 | M034L: synonymous |
| 35926 | 35934 | G | A | WS1 234 | M034L: synonymous |
| 36195 | 36127 | C | A | Meby | M034L: H222Y |
| 36430 | 36300 | G | C | BRK 12-2-93 | M034L: N143K |
| 36832 | 36689 | G | A | Gung | M034L: synonymous |
| 36832 | 36702 | G | A | BRK 12-2-93 | M034L: synonymous |
| 36832 | 36812 | G | A | Wellington | M034L: synonymous |
| 36832 | 37261 | G | A | BD23 | M034L: synonymous |
| 36832 | 36736 | G | A | BRK 897 | M034L: synonymous |
| 36832 | 36763 | G | A | OB1 406 | M034L: synonymous |
| 36832 | 36840 | G | A | WS1 234 | M034L: synonymous |
| 36832 | 36858 | G | A | WS6 1071 | M034L: synonymous |
| 36832 | 36702 | G | A | WS1 328 | M034L: synonymous |
| 36832 | 36687 | G | A | WS6 346 | M034L: synonymous |
| 36832 | 36829 | G | A | SWH 8-2-93 | M034L: synonymous |
| 36832 | 37512 | G | A | SWH 1209 | M034L: synonymous |
| 36832 | 36858 | G | A | BRK | M034L: synonymous |
| 36832 | 36856 | G | A | SWH | M034L: synonymous |
| 36832 | 36764 | G | A | Meby | M034L: synonymous |
| 36832 | 36828 | G | A | Bendigo | M034L: synonymous |
| 37363 | 37294 | G | A | OB1 406 | M036L:S628F |
| 37587 | 37457 | C | T | BRK 12-2-93 | M036L: synonymous |
| 37981 | 38410 | G | A | BD23 | M036L: A422V |
| 37981 | 38661 | G | A | SWH 1209 | M036L: A422V |
| 38369 | 38371 | A | G | Uriarra | M036L: F293L |
| 38414 | 38346 | G | A | Meby | M036L: P278S |
| 38437 | 38294 | C | T | Gung | M036L: C270Y |
| 38437 | 38307 | C | T | BRK 12-2-93 | M036L: C270Y |
| 38437 | 38417 | C | T | Wellington | M036L: C270Y |
| 38437 | 38866 | C | T | BD23 | M036L: C270Y |
| 38437 | 38341 | C | T | BRK 897 | M036L: C270Y |
| 38437 | 38368 | C | T | OB1 406 | M036L: C270Y |
| 38437 | 38445 | C | T | WS1 234 | M036L: C270Y |
| 38437 | 38463 | C | T | WS6 1071 | M036L: C270Y |
| 38437 | 38307 | C | T | WS1 328 | M036L: C270Y |
| 38437 | 38292 | C | T | WS6 346 | M036L: C270Y |
| 38437 | 38434 | C | T | SWH 8-2-93 | M036L: C270Y |
| 38437 | 39117 | C | T | SWH 1209 | M036L: C270Y |
| 38437 | 38463 | C | T | BRK | M036L: C270Y |
| 38437 | 38461 | C | T | SWH | M036L: C270Y |
| 38437 | 38369 | C | T | Meby | M036L: C270Y |
| 38437 | 38433 | C | T | Bendigo | M036L: C270Y |
| 38437 | 38440 | C | T | KM13 | M036L: C270Y |
| 38589-38680 | 38614 |  |  | BRK | M036L 92 nt deletion |
| 38679 | 38536 | C | T | Gung | M036L: synonymous |
| 38686 | 38620 | T | C | BRK | M036L: synonymous |
| 38873 | 38807 | C | T | BRK | M036L: E125K |
| 38987 | 38844 | G | A | Gung | M036L: synonymous |
| 38987 | 38857 | G | A | BRK 12-2-93 | M036L: synonymous |
| 38987 | 38967 | G | A | Wellington | M036L: synonymous |
| 38987 | 39416 | G | A | BD23 | M036L: synonymous |
| 38987 | 38891 | G | A | BRK 897 | M036L: synonymous |
| 38987 | 38918 | G | A | OB1 406 | M036L: synonymous |
| 38987 | 38995 | G | A | WS1 234 | M036L: synonymous |
| 38987 | 39013 | G | A | WS6 1071 | M036L: synonymous |
| 38987 | 38857 | G | A | WS1 328 | M036L: synonymous |
| 38987 | 38842 | G | A | WS6 346 | M036L: synonymous |
| 38987 | 38984 | G | A | SWH 8-2-93 | M036L: synonymous |
| 38987 | 39667 | G | A | SWH 1209 | M036L: synonymous |
| 38987 | 38921 | G | A | BRK | M036L: synonymous |
| 38987 | 39011 | G | A | SWH | M036L: synonymous |
| 38987 | 38919 | G | A | Meby | M036L: synonymous |
| 38987 | 38983 | G | A | Bendigo | M036L: synonymous |
| 40596 | 40593 | - | T | Bendigo | M040L: E258 insert |
| 40596 | 40594 | - | C | Bendigo | M040L: E258 insert |
| 40596 | 40595 | - | T | Bendigo | M040L: E258 insert |
| 41307 | 41164 | C | T | Gung | M040L: synonymous |
| 41334 | 41314 | C | T | Wellington | M040L: synonymous |
| 41406 | 41341 | - | T | BRK | intergenic |
| 41406 | 41431 | - | T | SWH | intergenic |
| 41406 | 41339 | - | T | Meby | intergenic |
| 41406 | 41406 | - | T | Bendigo | intergenic |
| 41406 | 41387 | - | T | Wellington | intergenic |
| 41406 | 41277 | - | T | BRK 12-2-93 | intergenic |
| 41406 | 41311 | - | T | BRK 897 | intergenic |
| 41406 | 41404 | - | T | SWH 8-2-93 | intergenic |
| 41406 | 42087 | - | T | SWH 1209 | intergenic |
| 41406 | 41338 | - | T | OB1 406 | intergenic |
| 41406 | 41262 | - | T | WS6 346 | intergenic |
| 41406 | 41415 | - | T | WS1 234 | intergenic |
| 41406 | 41277 | - | T | WS1 328 | intergenic |
| 41406 | 41433 | - | T | WS6 1071 | intergenic |
| 41406 | 41836 | - | T | BD23 | intergenic |
| 41534 | 41391 | A | G | Gung | M041L: V49A |
| 41563 | 41496 | C | T | Meby | M041L: synonymous |
| 41807 | 41834 | C | T | WS6 1071 | M042L: D352N |
| 42708 | 42689 | C | T | Wellington | M042L: synonymous |
| 42708 | 42613 | C | T | BRK 897 | M042L: synonymous |
| 42708 | 42640 | C | T | OB1 406 | M042L: synonymous |
| 42708 | 42717 | C | T | WS1 234 | M042L: synonymous |
| 42708 | 42579 | C | T | WS1 328 | M042L: synonymous |
| 42708 | 42706 | C | T | SWH 8-2-93 | M042L: synonymous |
| 42708 | 42643 | C | T | BRK | M042L: synonymous |
| 42708 | 42733 | C | T | SWH | M042L: synonymous |
| 42708 | 42708 | C | T | Bendigo | M042L: synonymous |
| 43248 | 43180 | C | T | OB1 406 | M043L: A299T |
| 43248 | 43119 | C | T | WS1 328 | M043L: A299T |
| 43248 | 43246 | C | T | SWH 8-2-93 | M043L: A299T |
| 43248 | 43183 | C | T | BRK | M043L: A299T |
| 43248 | 43273 | C | T | SWH | M043L: A299T |
| 43979 | 43911 | G | A | OB1 406 | M043L: A55V |
| 44965 | 44821 | T | C | WS6 346 | M044R: R273L |
| 45196 | 45128 | T | C | OB1 406 | M044R: V350A |
| 45196 | 45067 | T | C | WS1 328 | M044R: V350A |
| 45196 | 45194 | T | C | SWH 8-2-93 | M044R: V350A |
| 45196 | 45131 | T | C | BRK | M044R: V350A |
| 45196 | 45221 | T | C | SWH | M044R: V350A |
| 45229 | 45086 | C | T | Gung | M044R: S361L |
| 45229 | 45229 | C | T | Bendigo | M044R: S361L |
| 45944 | 45879 | C | T | BRK | M044R: synonymous |
| 45964 | 45951 | G | A | Glenfield | M044R: R606H |
| 46484 | 46465 | C | A | Wellington | M045L: synonymous |
| 46484 | 46389 | C | A | BRK 897 | M045L: synonymous |
| 46484 | 46493 | C | A | WS1 234 | M045L: synonymous |
| 47167 | 47024 | C | T | Gung | M045L: D263N |
| 47167 | 47038 | C | T | BRK 12-2-93 | M045L: D263N |
| 47167 | 47148 | C | T | Wellington | M045L: D263N |
| 47167 | 47597 | C | T | BD23 | M045L: D263N |
| 47167 | 47072 | C | T | BRK 897 | M045L: D263N |
| 47167 | 47099 | C | T | OB1 406 | M045L: D263N |
| 47167 | 47176 | C | T | WS1 234 | M045L: D263N |
| 47167 | 47194 | C | T | WS6 1071 | M045L: D263N |
| 47167 | 47038 | C | T | WS1 328 | M045L: D263N |
| 47167 | 47023 | C | T | WS6 346 | M045L: D263N |
| 47167 | 47165 | C | T | SWH 8-2-93 | M045L: D263N |
| 47167 | 47848 | C | T | SWH 1209 | M045L: D263N |
| 47167 | 47102 | C | T | BRK | M045L: D263N |
| 47167 | 47192 | C | T | SWH | M045L: D263N |
| 47167 | 47100 | C | T | Meby | M045L: D263N |
| 47167 | 47167 | C | T | Bendigo | M045L: D263N |
| 47635 | 48065 | A | G | BD23 | M045L: Y107H |
| 47635 | 48316 | A | G | SWH 1209 | M045L: Y107H |
| 48198 | 48196 | C | T | SWH 8-2-93 | M046L: E30K |
| 48622 | 48493 | C | T | BRK 12-2-93 | M047R: A115V |
| 48622 | 48649 | C | T | WS6 1071 | M047R: A115V |
| 48622 | 48478 | C | T | WS6 346 | M047R: A115V |
| 49216 | 49646 | C | T | BD23 | M048L: D29N |
| 49363 | 49344 | G | A | Wellington | M049R: E21K |
| 49674 | 49607 | G | T | Meby | M049R: synonymous |
| 49974 | 49955 | G | A | Wellington | M049R: synonymous |
| 49974 | 49879 | G | A | BRK 897 | M049R: synonymous |
| 49974 | 49983 | G | A | WS1 234 | M049R: synonymous |
| 49974 | 49974 | G | A | Bendigo | M049R: synonymous |
| 50242 | 50175 | A | G | Meby | M049R:T314A |
| 50304 | 50285 | G | A | Wellington | M049R: synon |
| 50435 | 50865 | C | T | BD23 | M049R: T378I |
| 50515 | 50372 | C | T | Gung | M049R: synonymous |
| 50515 | 50386 | C | T | BRK 12-2-93 | M049R: synonymous |
| 50515 | 50496 | C | T | Wellington | M049R: synonymous |
| 50515 | 50945 | C | T | BD23 | M049R: synonymous |
| 50515 | 50420 | C | T | BRK 897 | M049R: synonymous |
| 50515 | 50447 | C | T | OB1 406 | M049R: synonymous |
| 50515 | 50524 | C | T | WS1 234 | M049R: synonymous |
| 50515 | 50542 | C | T | WS6 1071 | M049R: synonymous |
| 50515 | 50386 | C | T | WS1 328 | M049R: synonymous |
| 50515 | 50371 | C | T | WS6 346 | M049R: synonymous |
| 50515 | 50513 | C | T | SWH 8-2-93 | M049R: synonymous |
| 50515 | 51196 | C | T | SWH 1209 | M049R: synonymous |
| 50515 | 50450 | C | T | BRK | M049R: synonymous |
| 50515 | 50540 | C | T | SWH | M049R: synonymous |
| 50515 | 50448 | C | T | Meby | M049R: synonymous |
| 50515 | 50515 | C | T | Bendigo | M049R: synonymous |
| 51200 | 51198 | C | A | SWH 8-2-93 | M051R: synonymous |
| 51473 | 51473 | T | C | Bendigo | M052L: D290G |
| 52059 | 52086 | A | G | WS6 1071 | M052L: synonymous |
| 52256 | 52113 | C | T | Gung | M052L: S29N |
| 52256 | 52127 | C | T | BRK 12-2-93 | M052L: S29N |
| 52256 | 52237 | C | T | Wellington | M052L: S29N |
| 52256 | 52686 | C | T | BD23 | M052L: S29N |
| 52256 | 52161 | C | T | BRK 897 | M052L: S29N |
| 52256 | 52188 | C | T | OB1 406 | M052L: S29N |
| 52256 | 52265 | C | T | WS1 234 | M052L: S29N |
| 52256 | 52283 | C | T | WS6 1071 | M052L: S29N |
| 52256 | 52127 | C | T | WS1 328 | M052L: S29N |
| 52256 | 52112 | C | T | WS6 346 | M052L: S29N |
| 52256 | 52254 | C | T | SWH 8-2-93 | M052L: S29N |
| 52256 | 52937 | C | T | SWH 1209 | M052L: S29N |
| 52256 | 52191 | C | T | BRK | M052L: S29N |
| 52256 | 52281 | C | T | SWH | M052L: S29N |
| 52256 | 52189 | C | T | Meby | M052L: S29N |
| 52256 | 52256 | C | T | Bendigo | M052L: S29N |
| 52278 | 52211 | T | C | Meby | M052L: T22A |
| 52751 | 52684 | C | T | Meby | M053R: synonymous |
| 53512 | 53369 | T | C | Gung | M054R: synonymous |
| 53512 | 53512 | T | C | Bendigo | M054R: synonymous |
| 53603 | 53474 | G | A | WS1 328 | M054R: A144T |
| 55900 | 55757 | A | C | Gung | M057L: L90V |
| 55900 | 55771 | A | C | BRK 12-2-93 | M057L: L90V |
| 55900 | 55881 | A | C | Wellington | M057L: L90V |
| 55900 | 56330 | A | C | BD23 | M057L: L90V |
| 55900 | 55805 | A | C | BRK 897 | M057L: L90V |
| 55900 | 55832 | A | C | OB1 406 | M057L: L90V |
| 55900 | 55909 | A | C | WS1 234 | M057L: L90V |
| 55900 | 55927 | A | C | WS6 1071 | M057L: L90V |
| 55900 | 55771 | A | C | WS1 328 | M057L: L90V |
| 55900 | 55756 | A | C | WS6 346 | M057L: L90V |
| 55900 | 55898 | A | C | SWH 8-2-93 | M057L: L90V |
| 55900 | 56581 | A | C | SWH 1209 | M057L: L90V |
| 55900 | 55835 | A | C | BRK | M057L: L90V |
| 55900 | 55925 | A | C | SWH | M057L: L90V |
| 55900 | 55833 | A | C | Meby | M057L: L90V |
| 55900 | 55900 | A | C | Bendigo | M057L: L90V |
| 56030 | 55887 | T | C | Gung | M057L: synonymous |
| 56030 | 56030 | T | C | Bendigo | M057L: synonymous |
| 56182 | 56115 | - | A | OB1 406 | intergenic |
| 57398 | 57255 | G | A | Gung | M060R: synonymous |
| 57398 | 57269 | G | A | BRK 12-2-93 | M060R: synonymous |
| 57398 | 57379 | G | A | Wellington | M060R: synonymous |
| 57398 | 57828 | G | A | BD23 | M060R: synonymous |
| 57398 | 57303 | G | A | BRK 897 | M060R: synonymous |
| 57398 | 57331 | G | A | OB1 406 | M060R: synonymous |
| 57398 | 57407 | G | A | WS1 234 | M060R: synonymous |
| 57398 | 57425 | G | A | WS6 1071 | M060R: synonymous |
| 57398 | 57269 | G | A | WS1 328 | M060R: synonymous |
| 57398 | 57254 | G | A | WS6 346 | M060R: synonymous |
| 57398 | 57396 | G | A | SWH 8-2-93 | M060R: synonymous |
| 57398 | 58079 | G | A | SWH 1209 | M060R: synonymous |
| 57398 | 57333 | G | A | BRK | M060R: synonymous |
| 57398 | 57423 | G | A | SWH | M060R: synonymous |
| 57398 | 57331 | G | A | Meby | M060R: synonymous |
| 57398 | 57398 | G | A | Bendigo | M060R: synonymous |
| 57976 | 57957 | A | G | Wellington | M061R: synonymous |
| 57976 | 57881 | A | G | BRK 897 | M061R: synonymous |
| 57976 | 57985 | A | G | WS1 234 | M061R: synonymous |
| 58264 | 58135 | C | T | BRK 12-2-93 | M061R: synonymous |
| 58264 | 58291 | C | T | WS6 1071 | M061R: synonymous |
| 58264 | 58120 | C | T | WS6 346 | M061R: synonymous |
| 58322 | 58332 | - | T | WS1 234 | M061R: read-through adds LKY |
| 58821 | 58678 | A | C | Gung | M062R:K142T |
| 58821 | 58692 | A | C | BRK 12-2-93 | M062R:K142T |
| 58821 | 58802 | A | C | Wellington | M062R:K142T |
| 58821 | 59251 | A | C | BD23 | M062R:K142T |
| 58821 | 58726 | A | C | BRK 897 | M062R:K142T |
| 58821 | 58754 | A | C | OB1 406 | M062R:K142T |
| 58821 | 58831 | A | C | WS1 234 | M062R:K142T |
| 58821 | 58848 | A | C | WS6 1071 | M062R:K142T |
| 58821 | 58692 | A | C | WS1 328 | M062R:K142T |
| 58821 | 58677 | A | C | WS6 346 | M062R:K142T |
| 58821 | 58819 | A | C | SWH 8-2-93 | M062R:K142T |
| 58821 | 59502 | A | C | SWH 1209 | M062R:K142T |
| 58821 | 58756 | A | C | BRK | M062R:K142T |
| 58821 | 58846 | A | C | SWH | M062R:K142T |
| 58821 | 58821 | A | C | Bendigo | M062R:K142T |
| 59034 | 58891 | A | G | Gung | M063R: synonymous |
| 59034 | 59034 | A | G | Bendigo | M063R: synonymous |
| 59143 | 59001 | - | A | Gung | M063R: H insert |
| 59143 | 59002 | - | C | Gung | M063R: H insert |
| 59143 | 59003 | - | C | Gung | M063R: H insert |
| 59230 | 59086 | C | T | WS6 346 | M063R: P101S |
| 59512 | 59372 | A | T | Gung | M063R: S195C |
| 59512 | 59383 | A | T | BRK 12-2-93 | M063R: S195C |
| 59512 | 59493 | A | T | Wellington | M063R: S195C |
| 59512 | 59942 | A | T | BD23 | M063R: S195C |
| 59512 | 59417 | A | T | BRK 897 | M063R: S195C |
| 59512 | 59445 | A | T | OB1 406 | M063R: S195C |
| 59512 | 59522 | A | T | WS1 234 | M063R: S195C |
| 59512 | 59539 | A | T | WS6 1071 | M063R: S195C |
| 59512 | 59383 | A | T | WS1 328 | M063R: S195C |
| 59512 | 59368 | A | T | WS6 346 | M063R: S195C |
| 59512 | 59510 | A | T | SWH 8-2-93 | M063R: S195C |
| 59512 | 60193 | A | T | SWH 1209 | M063R: S195C |
| 59512 | 59447 | A | T | BRK | M063R: S195C |
| 59512 | 59537 | A | T | SWH | M063R: S195C |
| 59512 | 59445 | A | T | Meby | M063R: S195C |
| 59512 | 59512 | A | T | Bendigo | M063R: S195C |
| 59554 | 59410 | G | - | WS6 346 | M063R: TEEEE deleted at C terminus |
| 59555 | 59410 | A | - | WS6 346 | M063R: TEEEE deleted at C terminus |
| 59556 | 59410 | C | - | WS6 346 | M063R: TEEEE deleted at C terminus |
| 59557 | 59410 | A | - | WS6 346 | M063R: TEEEE deleted at C terminus |
| 59558 | 59410 | C | - | WS6 346 | M063R: TEEEE deleted at C terminus |
| 59559 | 59410 | G | - | WS6 346 | M063R: TEEEE deleted at C terminus |
| 59560 | 59410 | G | - | WS6 346 | M063R: TEEEE deleted at C terminus |
| 59561 | 59410 | A | - | WS6 346 | M063R: TEEEE deleted at C terminus |
| 59562 | 59410 | G | - | WS6 346 | M063R: TEEEE deleted at C terminus |
| 59563 | 59410 | G | - | WS6 346 | M063R: TEEEE deleted at C terminus |
| 59564 | 59410 | A | - | WS6 346 | M063R: TEEEE deleted at C terminus |
| 59565 | 59410 | G | - | WS6 346 | M063R: TEEEE deleted at C terminus |
| 59566 | 59410 | G | - | WS6 346 | M063R: TEEEE deleted at C terminus |
| 59567 | 59410 | A | - | WS6 346 | M063R: TEEEE deleted at C terminus |
| 59568 | 59410 | A | - | WS6 346 | M063R: TEEEE deleted at C terminus |
| 59585 | 59444 | T | - | Gung | intergenic |
| 59585 | 59455 | T | - | BRK 12-2-93 | intergenic |
| 59585 | 59565 | T | - | Wellington | intergenic |
| 59585 | 60014 | T | - | BD23 | intergenic |
| 59585 | 59489 | T | - | BRK 897 | intergenic |
| 59585 | 59517 | T | - | OB1 406 | intergenic |
| 59585 | 59594 | T | - | WS1 234 | intergenic |
| 59585 | 59611 | T | - | WS6 1071 | intergenic |
| 59585 | 59455 | T | - | WS1 328 | intergenic |
| 59585 | 59425 | T | - | WS6 346 | intergenic |
| 59585 | 59582 | T | - | SWH 8-2-93 | intergenic |
| 59585 | 60265 | T | - | SWH 1209 | intergenic |
| 59585 | 59519 | T | - | BRK | intergenic |
| 59585 | 59609 | T | - | SWH | intergenic |
| 59585 | 59584 | T | - | Bendigo | intergenic |
| 60122 | 60053 | A | - | OB1 406 | M064R: in frame del E167 |
| 60123 | 60053 | G | - | OB1 406 | M064R: in frame del E167 |
| 60124 | 60053 | A | - | OB1 406 | M064R: in frame del E167 |
| 60122 | 59991 | A | - | BRK 12-2-93 | M064R: in frame del E167 |
| 60123 | 59991 | G | - | BRK 12-2-93 | M064R: in frame del E167 |
| 60124 | 59991 | A | - | BRK 12-2-93 | M064R: in frame del E167 |
| 60124 | 60105 | - | A | Wellington | M064R: in frame insert E168 |
| 60124 | 60106 | - | G | Wellington | M064R: in frame insert E168 |
| 60124 | 60107 | - | A | Wellington | M064R: in frame insert E168 |
| 60124 | 60029 | - | A | BRK 897 | M064R: in frame insert E168 |
| 60124 | 60030 | - | G | BRK 897 | M064R: in frame insert E168 |
| 60124 | 60031 | - | A | BRK 897 | M064R: in frame insert E168 |
| 60124 | 60134 | - | A | WS1 234 | M064R: in frame insert E168 |
| 60124 | 60135 | - | G | WS1 234 | M064R: in frame insert E168 |
| 60124 | 60136 | - | A | WS1 234 | M064R: in frame insert E168 |
| 60347 | 60214 | G | A | BRK 12-2-93 | M065R: E25K |
| 60347 | 60373 | G | A | WS6 1071 | M065R: E25K |
| 60347 | 60187 | G | A | WS6 346 | M065R: E25K |
| 60567 | 60426 | C | T | Gung | M065R: T98M |
| 60567 | 60434 | C | T | BRK 12-2-93 | M065R: T98M |
| 60567 | 60550 | C | T | Wellington | M065R: T98M |
| 60567 | 60996 | C | T | BD23 | M065R: T98M |
| 60567 | 60474 | C | T | BRK 897 | M065R: T98M |
| 60567 | 60496 | C | T | OB1 406 | M065R: T98M |
| 60567 | 60579 | C | T | WS1 234 | M065R: T98M |
| 60567 | 60593 | C | T | WS6 1071 | M065R: T98M |
| 60567 | 60437 | C | T | WS1 328 | M065R: T98M |
| 60567 | 60407 | C | T | WS6 346 | M065R: T98M |
| 60567 | 60564 | C | T | SWH 8-2-93 | M065R: T98M |
| 60567 | 61247 | C | T | SWH 1209 | M065R: T98M |
| 60567 | 60501 | C | T | BRK | M065R: T98M |
| 60567 | 60591 | C | T | SWH | M065R: T98M |
| 60567 | 60500 | C | T | Meby | M065R: T98M |
| 60567 | 60566 | C | T | Bendigo | M065R: T98M |
| 60663 | 60530 | C | T | BRK 12-2-93 | M065R: P130L |
| 60663 | 61092 | C | T | BD23 | M065R: P130L |
| 60663 | 60689 | C | T | WS6 1071 | M065R: P130L |
| 60663 | 60503 | C | T | WS6 346 | M065R: P130L |
| 60663 | 61343 | C | T | SWH 1209 | M065R: P130L |
| 61081 | 61014 | G | T | Meby | M065R: synonymous |
| 61657 | 61496 | G | A | Gung | M066R: synonymous |
| 62142 | 62009 | T | G | BRK 12-2-93 | M067L: E4A |
| 62142 | 62571 | T | G | BD23 | M067L: E4A |
| 62142 | 62168 | T | G | WS6 1071 | M067L: E4A |
| 62142 | 61982 | T | G | WS6 346 | M067L: E4A |
| 62142 | 62822 | T | G | SWH 1209 | M067L: E4A |
| 62171 | 62599 | A | - | BD23 | intergenic |
| 62562 | 62429 | C | T | BRK 12-2-93 | M068R: synonymous |
| 62562 | 62990 | C | T | BD23 | M068R: synonymous |
| 62562 | 62588 | C | T | WS6 1071 | M068R: synonymous |
| 62562 | 62402 | C | T | WS6 346 | M068R: synonymous |
| 62562 | 63242 | C | T | SWH 1209 | M068R: synonymous |
| 62661 | 62501 | G | A | WS6 346 | M068R: synonymous |
| 63209 | 63138 | C | G | OB1 406 | M068R: T328R |
| 63609 | 63538 | C | T | OB1 406 | M068R: synonymous |
| 64305 | 64164 | G | A | Gung | M068R: synonymous |
| 64305 | 64172 | G | A | BRK 12-2-93 | M068R: synonymous |
| 64305 | 64288 | G | A | Wellington | M068R: synonymous |
| 64305 | 64733 | G | A | BD23 | M068R: synonymous |
| 64305 | 64212 | G | A | BRK 897 | M068R: synonymous |
| 64305 | 64234 | G | A | OB1 406 | M068R: synonymous |
| 64305 | 64317 | G | A | WS1 234 | M068R: synonymous |
| 64305 | 64331 | G | A | WS6 1071 | M068R: synonymous |
| 64305 | 64175 | G | A | WS1 328 | M068R: synonymous |
| 64305 | 64145 | G | A | WS6 346 | M068R: synonymous |
| 64305 | 64302 | G | A | SWH 8-2-93 | M068R: synonymous |
| 64305 | 64985 | G | A | SWH 1209 | M068R: synonymous |
| 64305 | 64239 | G | A | BRK | M068R: synonymous |
| 64305 | 64329 | G | A | SWH | M068R: synonymous |
| 64305 | 64238 | G | A | Meby | M068R: synonymous |
| 64305 | 64304 | G | A | Bendigo | M068R: synonymous |
| 64305 | 64308 | G | A | KM13 | M068R: synonymous |
| 65261 | 65194 | A | G | Meby | M068R: D1012G |
| 67659 | 67661 | C | T | Uriarra | M071L: E172K |
| 67836 | 68264 | C | T | BD23 | M071L: V113I |
| 67836 | 67765 | C | T | OB1 406 | M071L: V113I |
| 67836 | 67706 | C | T | WS1 328 | M071L: V113I |
| 67836 | 67833 | C | T | SWH 8-2-93 | M071L: V113I |
| 67836 | 68516 | C | T | SWH 1209 | M071L: V113I |
| 67836 | 67770 | C | T | BRK | M071L: V113I |
| 67836 | 67860 | C | T | SWH | M071L: V113I |
| 68163 | 68030 | G | A | BRK 12-2-93 | M071L: P4S |
| 68163 | 68189 | G | A | WS6 1071 | M071L: P4S |
| 68163 | 68003 | G | A | WS6 346 | M071L: P4S |
| 70115 | 69974 | A | G | Gung | M072L: I150T |
| 70115 | 69982 | A | G | BRK 12-2-93 | M072L: I150T |
| 70115 | 70098 | A | G | Wellington | M072L: I150T |
| 70115 | 70543 | A | G | BD23 | M072L: I150T |
| 70115 | 70022 | A | G | BRK 897 | M072L: I150T |
| 70115 | 70044 | A | G | OB1 406 | M072L: I150T |
| 70115 | 70127 | A | G | WS1 234 | M072L: I150T |
| 70115 | 70141 | A | G | WS6 1071 | M072L: I150T |
| 70115 | 69985 | A | G | WS1 328 | M072L: I150T |
| 70115 | 69955 | A | G | WS6 346 | M072L: I150T |
| 70115 | 70112 | A | G | SWH 8-2-93 | M072L: I150T |
| 70115 | 70795 | A | G | SWH 1209 | M072L: I150T |
| 70115 | 70049 | A | G | BRK | M072L: I150T |
| 70115 | 70139 | A | G | SWH | M072L: I150T |
| 70115 | 70048 | A | G | Meby | M072L: I150T |
| 70115 | 70114 | A | G | Bendigo | M072L: I150T |
| 70115 | 70118 | A | G | KM13 | M072L: I150T |
| 70356 | 70223 | C | T | BRK 12-2-93 | M072L: D70N |
| 72844 | 72711 | G | A | BRK 12-2-93 | M076R: synonymous |
| 72844 | 72870 | G | A | WS6 1071 | M076R: synonymous |
| 72844 | 72684 | G | A | WS6 346 | M076R: synonymous |
| 72901 | 72741 | C | T | WS6 346 | M076R: synonymous |
| 75882 | 75816 | C | A | BRK | M078R: synonymous |
| 80096 | 80079 | C | T | Wellington | M081R: R234C |
| 80096 | 80003 | C | T | BRK 897 | M081R: R234C |
| 80096 | 80025 | C | T | OB1 406 | M081R: R234C |
| 80096 | 80108 | C | T | WS1 234 | M081R: R234C |
| 80524 | 80453 | C | T | OB1 406 | M081R: synonymous |
| 80524 | 80394 | C | T | WS1 328 | M081R: synonymous |
| 80524 | 80521 | C | T | SWH 8-2-93 | M081R: synonymous |
| 80524 | 80458 | C | T | BRK | M081R: synonymous |
| 80524 | 80548 | C | T | SWH | M081R: synonymous |
| 81865 | 81867 | G | A | Uriarra | M081R: synonymous |
| 82120 | 81980 | - | G | Gung | M083L: corrects orf |
| 82120 | 82120 | - | G | Bendigo | M083L: corrects orf |
| 82120 | 81988 | - | G | BRK 12-2-93 | M083L: corrects orf |
| 82120 | 82104 | - | G | Wellington | M083L: corrects orf |
| 82120 | 82549 | - | G | BD23 | M083L: corrects orf |
| 82120 | 82028 | - | G | BRK 897 | M083L: corrects orf |
| 82120 | 82050 | - | G | OB1 406 | M083L: corrects orf |
| 82120 | 82133 | - | G | WS1 234 | M083L: corrects orf |
| 82120 | 82147 | - | G | WS6 1071 | M083L: corrects orf |
| 82120 | 81991 | - | G | WS1 328 | M083L: corrects orf |
| 82120 | 81961 | - | G | WS6 346 | M083L: corrects orf |
| 82120 | 82118 | - | G | SWH 8-2-93 | M083L: corrects orf |
| 82120 | 82801 | - | G | SWH 1209 | M083L: corrects orf |
| 82120 | 82055 | - | G | BRK | M083L: corrects orf |
| 82120 | 82145 | - | G | SWH | M083L: corrects orf |
| 82120 | 82054 | - | G | Meby | M083L: corrects orf |
| 82964 | 82964 | C | T | Bendigo | M084R: synonymous |
| 82985 | 82893 | T | C | BRK 897 | M084R: synonymous |
| 82985 | 82998 | T | C | WS1 234 | M084R: synonymous |
| 83002 | 83002 | C | T | Bendigo | M084R: S109L |
| 83354 | 83214 | T | C | Gung | M085R: synonymous |
| 85037 | 84878 | G | A | WS6 346 | M086L: synonymous |
| 85496 | 85356 | T | C | Gung | M086L: synonymous |
| 85496 | 85364 | T | C | BRK 12-2-93 | M086L: synonymous |
| 85496 | 85480 | T | C | Wellington | M086L: synonymous |
| 85496 | 85925 | T | C | BD23 | M086L: synonymous |
| 85496 | 85404 | T | C | BRK 897 | M086L: synonymous |
| 85496 | 85426 | T | C | OB1 406 | M086L: synonymous |
| 85496 | 85509 | T | C | WS1 234 | M086L: synonymous |
| 85496 | 85523 | T | C | WS6 1071 | M086L: synonymous |
| 85496 | 85367 | T | C | WS1 328 | M086L: synonymous |
| 85496 | 85337 | T | C | WS6 346 | M086L: synonymous |
| 85496 | 85494 | T | C | SWH 8-2-93 | M086L: synonymous |
| 85496 | 86177 | T | C | SWH 1209 | M086L: synonymous |
| 85496 | 85431 | T | C | BRK | M086L: synonymous |
| 85496 | 85521 | T | C | SWH | M086L: synonymous |
| 85496 | 85430 | T | C | Meby | M086L: synonymous |
| 85496 | 85496 | T | C | Bendigo | M086L: synonymous |
| 86756 | 86781 | G | A | SWH | M087L: P33L |
| 89461 | 90142 | C | T | SWH 1209 | M090L: synonymous |
| 91460 | 91320 | C | T | Gung | M092L: synonymous |
| 91460 | 91444 | C | T | Wellington | M092L: synonymous |
| 91460 | 91368 | C | T | BRK 897 | M092L: synonymous |
| 91460 | 91473 | C | T | WS1 234 | M092L: synonymous |
| 91460 | 91460 | C | T | Bendigo | M092L: synonymous |
| 91559 | 91493 | C | T | Meby | M092L: M119I |
| 91835 | 91862 | C | T | WS6 1071 | M092L: synonymous |
| 92163 | 92189 | - | G | SWH | M093L: VP insert |
| 92163 | 92190 | - | G | SWH | M093L: VP insert |
| 92163 | 92191 | - | G | SWH | M093L: VP insert |
| 92163 | 92192 | - | A | SWH | M093L: VP insert |
| 92163 | 92193 | - | A | SWH | M093L: VP insert |
| 92163 | 92194 | - | C | SWH | M093L: VP insert |
| 95536 | 96217 | C | T | SWH 1209 | M096L:D237N |
| 99159 | 99146 | C | T | Glenfield | M099L: synonymous |
| 99168 | 99028 | C | T | Gung | M099L: synonymous |
| 99168 | 99036 | C | T | BRK 12-2-93 | M099L: synonymous |
| 99168 | 99152 | C | T | Wellington | M099L: synonymous |
| 99168 | 99597 | C | T | BD23 | M099L: synonymous |
| 99168 | 99076 | C | T | BRK 897 | M099L: synonymous |
| 99168 | 99098 | C | T | OB1 406 | M099L: synonymous |
| 99168 | 99181 | C | T | WS1 234 | M099L: synonymous |
| 99168 | 99195 | C | T | WS6 1071 | M099L: synonymous |
| 99168 | 99039 | C | T | WS1 328 | M099L: synonymous |
| 99168 | 99009 | C | T | WS6 346 | M099L: synonymous |
| 99168 | 99166 | C | T | SWH 8-2-93 | M099L: synonymous |
| 99168 | 99849 | C | T | SWH 1209 | M099L: synonymous |
| 99168 | 99103 | C | T | BRK | M099L: synonymous |
| 99168 | 99199 | C | T | SWH | M099L: synonymous |
| 99168 | 99168 | C | T | Bendigo | M099L: synonymous |
| 99188 | 99172 | A | G | Wellington | M099L: Y302H |
| 99188 | 99096 | A | G | BRK 897 | M099L: Y302H |
| 99188 | 99201 | A | G | WS1 234 | M099L: Y302H |
| 99188 | 99188 | A | G | Bendigo | M099L: Y302H |
| 99240 | 99175 | G | A | BRK | M099L: synonymous |
| 99258 | 99242 | G | A | Wellington | M099L: synonymous |
| 99411 | 99279 | C | T | BRK 12-2-93 | M099L: synonymous |
| 99411 | 99438 | C | T | WS6 1071 | M099L: synonymous |
| 99411 | 99252 | C | T | WS6 346 | M099L: synonymous |
| 101819 | 101803 | A | G | Wellington | intergenic |
| 101819 | 101727 | A | G | BRK 897 | intergenic |
| 101819 | 101832 | A | G | WS1 234 | intergenic |
| 101823 | 101757 | G | A | Meby | intergenic |
| 101965 | 101992 | T | C | WS6 1071 | M103L: T51A |
| 102017 | 101952 | G | A | BRK | M103L: synonymous |
| 102310 | 102337 | C | T | WS6 1071 | M103L: synonymous |
| 102541 | 102525 | G | A | Wellington | M103L: synonymous |
| 102541 | 102449 | G | A | BRK 897 | M103L: synonymous |
| 102541 | 102554 | G | A | WS1 234 | M103L: synonymous |
| 102662 | 102530 | G | A | BRK 12-2-93 | M103L: synonymous |
| 102662 | 103091 | G | A | BD23 | M103L: synonymous |
| 102662 | 102689 | G | A | WS6 1071 | M103L: synonymous |
| 102662 | 102503 | G | A | WS6 346 | M103L: synonymous |
| 102662 | 103343 | G | A | SWH 1209 | M103L: synonymous |
| 103274 | 103182 | C | T | BRK 897 | M106L: M136I |
| 103274 | 103287 | C | T | WS1 234 | M106L: M136I |
| 103229 | 103189 | G | A | Gung | M106L: M136I |
| 103786 | 103720 | C | T | Meby | M107L: A172T |
| 103919 | 104348 | G | A | BD23 | M107L: synonymous |
| 104365 | 104352 | T | A | Glenfield | M108R: F18I |
| 104425 | 104409 | C | T | Wellington | M108R: P38S |
| 104425 | 104333 | C | T | BRK 897 | M108R: P38S |
| 104425 | 104438 | C | T | WS1 234 | M108R: P38S |
| 106040 | 105974 | G | A | Meby | M110L: synonymous |
| 107096 | 107123 | G | A | WS6 1071 | M110L: synonymous |
| 107678 | 107613 | C | T | BRK | M112R: A47V |
| 109894 | 110575 | G | A | SWH 1209 | M114R: synonymous |
| 111300 | 111287 | C | T | Glenfield | M114R: A686V |
| 111433 | 111417 | C | T | Wellington | M114R: synonymous |
| 111433 | 111341 | C | T | BRK 897 | M114R: synonymous |
| 111433 | 111446 | C | T | WS1 234 | M114R: synonymous |
| 111433 | 111433 | C | T | Bendigo | M114R: synonymous |
| 112683 | 112551 | C | A | BRK 12-2-93 | M114R: P1147H |
| 112683 | 113112 | C | A | BD23 | M114R: P1147H |
| 112683 | 112613 | C | A | OB1 406 | M114R: P1147H |
| 112683 | 112554 | C | A | WS1 328 | M114R: P1147H |
| 112683 | 112524 | C | A | WS6 346 | M114R: P1147H |
| 112683 | 112681 | C | A | SWH 8-2-93 | M114R: P1147H |
| 112683 | 113364 | C | A | SWH 1209 | M114R: P1147H |
| 112683 | 112618 | C | A | BRK | M114R: P1147H |
| 112683 | 112714 | C | A | SWH | M114R: P1147H |
| 112683 | 112683 | C | A | Bendigo | M114R: P1147H |
| 113080 | 112939 | C | - | Gung | M115L: D66, E67 deleted |
| 113081 | 112939 | T | - | Gung | M115L: D66, E67 deleted |
| 113082 | 112939 | T | - | Gung | M115L: D66, E67 deleted |
| 113083 | 112939 | C | - | Gung | M115L: D66, E67 deleted |
| 113084 | 112939 | G | - | Gung | M115L: D66, E67 deleted |
| 113085 | 112939 | T | - | Gung | M115L: D66, E67 deleted |
| 113089 | 113518 | G | A | BD23 | M115L: P65S |
| 113313 | 113181 | C | T | BRK 12-2-93 | M116L: R131K |
| 115902 | 115756 | C | T | Gung | M121R: S21F |
| 115902 | 115770 | C | T | BRK 12-2-93 | M121R: S21F |
| 115902 | 115887 | C | T | Wellington | M121R: S21F |
| 115902 | 116331 | C | T | BD23 | M121R: S21F |
| 115902 | 115810 | C | T | BRK 897 | M121R: S21F |
| 115902 | 115832 | C | T | OB1 406 | M121R: S21F |
| 115902 | 115915 | C | T | WS1 234 | M121R: S21F |
| 115902 | 115929 | C | T | WS6 1071 | M121R: S21F |
| 115902 | 115773 | C | T | WS1 328 | M121R: S21F |
| 115902 | 115743 | C | T | WS6 346 | M121R: S21F |
| 115902 | 115900 | C | T | SWH 8-2-93 | M121R: S21F |
| 115902 | 116583 | C | T | SWH 1209 | M121R: S21F |
| 115902 | 115837 | C | T | BRK | M121R: S21F |
| 115902 | 115933 | C | T | SWH | M121R: S21F |
| 115902 | 115902 | C | T | Bendigo | M121R: S21F |
| 116797 | 116797 | T | C | Bendigo | M122R: synonymous |
| 116914 | 116897 | T | - | Wellington | intergenic |
| 116914 | 116821 | T | - | BRK 897 | intergenic |
| 116914 | 116926 | T | - | WS1 234 | intergenic |
| 116914 | 116911 | T | - | SWH 8-2-93 | intergenic |
| 116914 | 116913 | T | . | Bendigo | intergenic |
| 119939 | 119922 | G | A | Wellington | M127L: synonymous |
| 119939 | 120368 | G | A | BD23 | M127L: synonymous |
| 119939 | 119846 | G | A | BRK 897 | M127L: synonymous |
| 119939 | 119869 | G | A | OB1 406 | M127L: synonymous |
| 119939 | 119951 | G | A | WS1 234 | M127L: synonymous |
| 119939 | 119810 | G | A | WS1 328 | M127L: synonymous |
| 119939 | 119936 | G | A | SWH 8-2-93 | M127L: synonymous |
| 119939 | 120620 | G | A | SWH 1209 | M127L: synonymous |
| 119939 | 119874 | G | A | BRK | M127L: synonymous |
| 119939 | 119970 | G | A | SWH | M127L: synonymous |
| 119939 | 119938 | G | A | Bendigo | M127L: synonymous |
| 120019 | 120018 | C | T | Bendigo | M127L: A343T |
| 122338 | 122192 | C | A | Gung | intergenic |
| 122338 | 122365 | C | A | WS6 1071 | intergenic |
| 122397 | 122385 | - | G | Glenfield | M130R: frameshift |
| 123337 | 123364 | A | G | WS6 1071 | M132L: V157A |
| 123409 | 123263 | C | T | Gung | M132L: C133Y |
| 123409 | 123392 | C | T | Wellington | M132L: C133Y |
| 123409 | 123838 | C | T | BD23 | M132L: C133Y |
| 123409 | 123316 | C | T | BRK 897 | M132L: C133Y |
| 123409 | 123339 | C | T | OB1 406 | M132L: C133Y |
| 123409 | 123421 | C | T | WS1 234 | M132L: C133Y |
| 123409 | 123280 | C | T | WS6 1071 | M132L: C133Y |
| 123409 | 123436 | C | T | WS1 328 | M132L: C133Y |
| 123409 | 123406 | C | T | SWH 8-2-93 | M132L: C133Y |
| 123409 | 124090 | C | T | SWH 1209 | M132L: C133Y |
| 123409 | 123344 | C | T | BRK | M132L: C133Y |
| 123409 | 123440 | C | T | SWH | M132L: C133Y |
| 123409 | 123408 | C | T | Bendigo | M132L: C133Y |
| 123729 | 123664 | C | T | BRK | M132L: synonymous |
| 123779 | 123776 | T | C | SWH 8-2-93 | M132L: T10A |
| 124945 | 124879 | G | A | Meby | M133R: synonymous |
| 125826 | 125680 | G | A | Gung | M133R: synonymous |
| 125826 | 125853 | G | A | WS6 1071 | M133R: synonymous |
| 125826 | 125667 | G | A | WS6 346 | M133R: synonymous |
| 125935 | 125789 | T | C | Gung | M134R: S84P |
| 125935 | 125803 | T | C | BRK 12-2-93 | M134R: S84P |
| 125935 | 125918 | T | C | Wellington | M134R: S84P |
| 125935 | 126364 | T | C | BD23 | M134R: S84P |
| 125935 | 125842 | T | C | BRK 897 | M134R: S84P |
| 125935 | 125865 | T | C | OB1 406 | M134R: S84P |
| 125935 | 125947 | T | C | WS1 234 | M134R: S84P |
| 125935 | 125962 | T | C | WS6 1071 | M134R: S84P |
| 125935 | 125806 | T | C | WS1 328 | M134R: S84P |
| 125935 | 125776 | T | C | WS6 346 | M134R: S84P |
| 125935 | 125932 | T | C | SWH 8-2-93 | M134R: S84P |
| 125935 | 126616 | T | C | SWH 1209 | M134R: S84P |
| 125935 | 125870 | T | C | BRK | M134R: S84P |
| 125935 | 125966 | T | C | SWH | M134R: S84P |
| 125935 | 125869 | T | C | Meby | M134R: S84P |
| 125935 | 125934 | T | C | Bendigo | M134R: S84P |
| 126068 | 125936 | C | T | BRK 12-2-93 | M134R: S128F |
| 126068 | 125909 | C | T | WS6 346 | M134R: S128F |
| 126071 | 126001 | T | C | OB1 406 | M134R: V129A |
| 127484 | 127338 | G | A | Gung | M134R: R600K |
| 127484 | 127511 | G | A | WS6 1071 | M134R: R600K |
| 127515 | 127512 | G | A | SWH 8-2-93 | M134R: synonymous |
| 128111 | 127979 | A | G | BRK 12-2-93 | M134R: K809R |
| 128748 | 128602 | G | A | Gung | M134R: synonymous |
| 128748 | 128616 | G | A | BRK 12-2-93 | M134R: synonymous |
| 128748 | 128731 | G | A | Wellington | M134R: synonymous |
| 128748 | 129177 | G | A | BD23 | M134R: synonymous |
| 128748 | 128655 | G | A | BRK 897 | M134R: synonymous |
| 128748 | 128678 | G | A | OB1 406 | M134R: synonymous |
| 128748 | 128760 | G | A | WS1 234 | M134R: synonymous |
| 128748 | 128775 | G | A | WS6 1071 | M134R: synonymous |
| 128748 | 128619 | G | A | WS1 328 | M134R: synonymous |
| 128748 | 128589 | G | A | WS6 346 | M134R: synonymous |
| 128748 | 128745 | G | A | SWH 8-2-93 | M134R: synonymous |
| 128748 | 129429 | G | A | SWH 1209 | M134R: synonymous |
| 128748 | 128683 | G | A | BRK | M134R: synonymous |
| 128748 | 128779 | G | A | SWH | M134R: synonymous |
| 128748 | 128682 | G | A | Meby | M134R: synonymous |
| 128748 | 128747 | G | A | Bendigo | M134R: synonymous |
| 130973 | 130907 | A | C | Meby | M134R: E1763A |
| 131579 | 132260 | G | A | SWH 1209 | M134R: R1965H |
| 131595 | 131531 | - | AAA | BRK | M134R: K insert |
| 131595 | 131628 | - | AAA | SWH | M134R: K insert |
| 131595 | 131530 | - | AAA | Meby | M134R: K insert |
| 131595 | 131595 | - | AAA | Bendigo | M134R: K insert |
| 131595 | 131598 | - | A | Uriarra | M134R : frameshift |
| 131595 | 131451 | - | AAA | Gung | M134R: K insert |
| 131595 | 131464 | - | AAA | BRK 12-2-93 | M134R: K insert |
| 131595 | 131579 | - | AAA | Wellington | M134R: K insert |
| 131595 | 132025 | - | AAA | BD23 | M134R: K insert |
| 131595 | 131503 | - | AAA | BRK 897 | M134R: K insert |
| 131595 | 131526 | - | AAA | OB1 406 | M134R: K insert |
| 131595 | 131608 | - | AAA | WS1 234 | M134R: K insert |
| 131595 | 131623 | - | AAA | WS6 1071 | M134R: K insert |
| 131595 | 131467 | - | AAA | WS1 328 | M134R: K insert |
| 131595 | 131437 | - | AAA | WS6 346 | M134R: K insert |
| 131595 | 131593 | - | AAA | SWH 8-2-93 | M134R: K insert |
| 131595 | 132277 | - | AAA | SWH 1209 | M134R: K insert |
| 131595 | 131584 | - | AAA | Glenfield | M134R: K insert |
| 131595 | 131599 | - | AAA | KM13 | M134R: K insert |
| 132910 | 133342 | G | A | BD23 | M137R: R4H |
| 132910 | 133594 | G | A | SWH 1209 | M137R: R4H |
| 133151 | 133008 | C | T | Gung | M137R: synonymous |
| 133151 | 133022 | C | T | BRK 12-2-93 | M137R: synonymous |
| 133151 | 133137 | C | T | Wellington | M137R: synonymous |
| 133151 | 133583 | C | T | BD23 | M137R: synonymous |
| 133151 | 133061 | C | T | BRK 897 | M137R: synonymous |
| 133151 | 133084 | C | T | OB1 406 | M137R: synonymous |
| 133151 | 133151 | C | T | SWH 8-2-93 | M137R: synonymous |
| 133151 | 133166 | C | T | WS1 234 | M137R: synonymous |
| 133151 | 133181 | C | T | WS6 1071 | M137R: synonymous |
| 133151 | 133025 | C | T | WS1 328 | M137R: synonymous |
| 133151 | 132995 | C | T | WS6 346 | M137R: synonymous |
| 133151 | 133835 | C | T | SWH 1209 | M137R: synonymous |
| 133151 | 133090 | C | T | BRK | M137R: synonymous |
| 133151 | 133185 | C | T | SWH | M137R: synonymous |
| 133151 | 133153 | C | T | Bendigo | M137R: synonymous |
| 133191 | 133124 | G | A | OB1 406 | M137R: D98N |
| 133500 | 133502 | G | A | Bendigo | M137R: A201T |
| 133539 | 133410 | G | T | BRK 12-2-93 | M137R: D214C |
| 133821 | 133824 | G | A | Uriarra | M137R: A308T |
| 134666 | 134668 | T | G | Bendigo | M138L: M25L |
| 134761 | 134762 | - | T | SWH 8-2-93 | intergenic |
| 135007 | 134864 | G | A | Gung | M139R: synonymous |
| 135007 | 135037 | G | A | WS6 1071 | M139R: synonymous |
| 135007 | 135009 | G | A | Bendigo | M139R: synonymous |
| 135127 | 135064 | G | T | Meby | M139R: R210S |
| 135593 | 135450 | C | A | Gung | M140R: P76H |
| 135593 | 135464 | C | A | BRK 12-2-93 | M140R: P76H |
| 135593 | 135579 | C | A | Wellington | M140R: P76H |
| 135593 | 136025 | C | A | BD23 | M140R: P76H |
| 135593 | 135503 | C | A | BRK 897 | M140R: P76H |
| 135593 | 135526 | C | A | OB1 406 | M140R: P76H |
| 135593 | 135608 | C | A | WS1 234 | M140R: P76H |
| 135593 | 135623 | C | A | WS6 1071 | M140R: P76H |
| 135593 | 135467 | C | A | WS1 328 | M140R: P76H |
| 135593 | 135437 | C | A | WS6 346 | M140R: P76H |
| 135593 | 135594 | C | A | SWH 8-2-93 | M140R: P76H |
| 135593 | 136277 | C | A | SWH 1209 | M140R: P76H |
| 135593 | 135584 | C | A | Glenfield | M140R: P76H |
| 135593 | 135531 | C | A | BRK | M140R: P76H |
| 135593 | 135627 | C | A | SWH | M140R: P76H |
| 135593 | 135530 | C | A | Meby | M140R: P76H |
| 135593 | 135595 | C | A | Bendigo | M140R: P76H |
| 135593 | 135599 | C | A | KM13 | M140R: P76H |
| 135593 | 135596 | C | A | Uriarra | M140R: P76H |
| 136251 | 136683 | C | T | BD23 | M140R: synonymous |
| 136449 | 136386 | G | A | Meby | M140R: synonymous |
| 136474 | 136411 | A | G | Meby | M140R: T371A |
| 137195 | 137187 | - | A | Glenfield | M141R: S insert |
| 137195 | 137188 | - | G | Glenfield | M141R: S insert |
| 137195 | 137189 | - | T | Glenfield | M141R: S insert |
| 137636 | 138068 | A | G | BD23 | M141R: synonymous |
| 137636 | 138320 | A | G | SWH 1209 | M141R: synonymous |
| 138270 | 138272 | A | G | Bendigo | M142R:Y183C |
| 139200 | 139230 | G | A | WS6 1071 | M143R:A182T |
| 139401 | 139258 | C | T | Gung | intergenic |
| 139486 | 139357 | G | A | BRK 12-2-93 | M144R: synonymous |
| 139719 | 139590 | A | G | BRK 12-2-93 | M144R: D106G |
| 139719 | 139705 | A | G | Wellington | M144R: D106G |
| 139719 | 139629 | A | G | BRK 897 | M144R: D106G |
| 139719 | 139734 | A | G | WS1 234 | M144R: D106G |
| 139719 | 139749 | A | G | WS6 1071 | M144R: D106G |
| 139719 | 139563 | A | G | WS6 346 | M144R: D106G |
| 139719 | 139720 | A | G | SWH 8-2-93 | M144R: D106G |
| 140451 | 140384 | A | G | OB1 406 | M146R: E42G |
| 141092 | 141523 | G | - | BD23 | M147R: frameshift |
| 141092 | 141523 | T | - | BD23 | M147R: frameshift |
| 141574 | 141511 | T | - | BRK | intergenic |
| 141574 | 141607 | T | - | SWH | intergenic |
| 141574 | 142003 | T | - | BD23 | intergenic |
| 141574 | 141506 | T | - | OB1 406 | intergenic |
| 141574 | 141447 | T | - | WS1 328 | intergenic |
| 141574 | 141257 | T | - | SWH 1209 | intergenic |
| 141739 | 141725 | G | A | Wellington | M148R: R41Q |
| 141739 | 141649 | G | A | BRK 897 | M148R: R41Q |
| 141739 | 141754 | G | A | WS1 234 | M148R: R41Q |
| 141739 | 141740 | G | A | SWH 8-2-93 | M148R: R41Q |
| 141832 | 141703 | C | T | BRK 12-2-93 | M148R: A72V |
| 141832 | 141862 | C | T | WS6 1071 | M148R: A72V |
| 141832 | 141676 | C | T | WS6 346 | M148R: A72V |
| 142595 | 142532 | C | T | BRK | M148R: synonymous |
| 142764 | 142621 | C | T | Gung | M148R: L383F |
| 142764 | 142635 | C | T | BRK 12-2-93 | M148R: L383F |
| 142764 | 142750 | C | T | Wellington | M148R: L383F |
| 142764 | 143193 | C | T | BD23 | M148R: L383F |
| 142764 | 142674 | C | T | BRK 897 | M148R: L383F |
| 142764 | 142696 | C | T | OB1 406 | M148R: L383F |
| 142764 | 142779 | C | T | WS1 234 | M148R: L383F |
| 142764 | 142794 | C | T | WS6 1071 | M148R: L383F |
| 142764 | 142637 | C | T | WS1 328 | M148R: L383F |
| 142764 | 142608 | C | T | WS6 346 | M148R: L383F |
| 142764 | 142765 | C | T | SWH 8-2-93 | M148R: L383F |
| 142764 | 143447 | C | T | SWH 1209 | M148R: L383F |
| 142764 | 142701 | C | T | BRK | M148R: L383F |
| 142764 | 142797 | C | T | SWH | M148R: L383F |
| 142764 | 142701 | C | T | Meby | M148R: L383F |
| 142764 | 142766 | C | T | Bendigo | M148R: L383F |
| 145699 | 145556 | C | T | Gung | M150R: P173S |
| 145699 | 145570 | C | T | BRK 12-2-93 | M150R: P173S |
| 145699 | 145685 | C | T | Wellington | M150R: P173S |
| 145699 | 146128 | C | T | BD23 | M150R: P173S |
| 145699 | 145609 | C | T | BRK 897 | M150R: P173S |
| 145699 | 145631 | C | T | OB1 406 | M150R: P173S |
| 145699 | 145714 | C | T | WS1 234 | M150R: P173S |
| 145699 | 145729 | C | T | WS6 1071 | M150R: P173S |
| 145699 | 145572 | C | T | WS1 328 | M150R: P173S |
| 145699 | 145543 | C | T | WS6 346 | M150R: P173S |
| 145699 | 145700 | C | T | SWH 8-2-93 | M150R: P173S |
| 145699 | 146382 | C | T | SWH 1209 | M150R: P173S |
| 145699 | 145636 | C | T | BRK | M150R: P173S |
| 145699 | 145732 | C | T | SWH | M150R: P173S |
| 145699 | 145636 | C | T | Meby | M150R: P173S |
| 145699 | 145701 | C | T | Bendigo | M150R: P173S |
| 147093 | 146950 | C | T | Gung | M151R: P140S |
| 147093 | 147095 | C | T | Bendigo | M151R: P140S |
| 147192 | 147049 | A | G | Gung | M151R: R173G |
| 147192 | 147063 | A | G | BRK 12-2-93 | M151R: R173G |
| 147192 | 147178 | A | G | Wellington | M151R: R173G |
| 147192 | 147621 | A | G | BD23 | M151R: R173G |
| 147192 | 147102 | A | G | BRK 897 | M151R: R173G |
| 147192 | 147124 | A | G | OB1 406 | M151R: R173G |
| 147192 | 147207 | A | G | WS1 234 | M151R: R173G |
| 147192 | 147222 | A | G | WS6 1071 | M151R: R173G |
| 147192 | 147065 | A | G | WS1 328 | M151R: R173G |
| 147192 | 147036 | A | G | WS6 346 | M151R: R173G |
| 147192 | 147193 | A | G | SWH 8-2-93 | M151R: R173G |
| 147192 | 147875 | A | G | SWH 1209 | M151R: R173G |
| 147192 | 147129 | A | G | BRK | M151R: R173G |
| 147192 | 147225 | A | G | SWH | M151R: R173G |
| 147192 | 147129 | A | G | Meby | M151R: R173G |
| 147192 | 147194 | A | G | Bendigo | M151R: R173G |
| 147865 | 147802 | T | C | Meby | M152R: synonymous |
| 147996 | 147853 | T | C | Gung | M152R; V106A |
| 148221 | 148207 | G | A | Wellington | M152R: R181H |
| 148485 | 148514 | A | - | WS6 1071 | M152R: early stop |
| 148635 | 148492 | G | T | Gung | M153R: R40L |
| 148711 | 148582 | T | C | BRK 12-2-93 | M153R: synonymous |
| 148711 | 148697 | T | C | Wellington | M153R: synonymous |
| 148711 | 148621 | T | C | BRK 897 | M153R: synonymous |
| 148711 | 148643 | T | C | OB1 406 | M153R: synonymous |
| 148711 | 148726 | T | C | WS1 234 | M153R: synonymous |
| 148711 | 148740 | T | C | WS6 1071 | M153R: synonymous |
| 148711 | 148584 | T | C | WS1 328 | M153R: synonymous |
| 148711 | 148555 | T | C | WS6 346 | M153R: synonymous |
| 148711 | 149140 | T | C | BD23 | M153R: synonymous |
| 148711 | 148712 | T | C | SWH 8-2-93 | M153R: synonymous |
| 148711 | 148648 | T | C | BRK | M153R: synonymous |
| 148711 | 148713 | T | C | Bendigo | M153R: synonymous |
| 148845 | 148838 | G | - | Glenfield | M153R: frameshift |
| 148985 | 149013 | T | - | WS6 1071 | M153R: frameshift |
| 149018 | 148954 |  | 73 nt deletion | Meby | M153R: frameshift/read-through |
| 149062 | 149047 | G | - | Wellington | M153R: VEE 182-184 deletion |
| 149063 | 149047 | G | - | Wellington | M153R: VEE 182-184 deletion |
| 149064 | 149047 | A | - | Wellington | M153R: VEE 182-184 deletion |
| 149065 | 149047 | G | - | Wellington | M153R: VEE 182-184 deletion |
| 149066 | 149047 | G | - | Wellington | M153R: VEE 182-184 deletion |
| 149067 | 149047 | A | - | Wellington | M153R: VEE 182-184 deletion |
| 149068 | 149047 | G | - | Wellington | M153R: VEE 182-184 deletion |
| 149069 | 149047 | G | - | Wellington | M153R: VEE 182-184 deletion |
| 149070 | 149047 | T | - | Wellington | M153R: VEE 182-184 deletion |
| 149127 | 148984 | T | C | Gung | M153R: L204S |
| 149127 | 148998 | T | C | BRK 12-2-93 | M153R: L204S |
| 149127 | 149104 | T | C | Wellington | M153R: L204S |
| 149127 | 149556 | T | C | BD23 | M153R: L204S |
| 149127 | 149037 | T | C | BRK 897 | M153R: L204S |
| 149127 | 149059 | T | C | OB1 406 | M153R: L204S |
| 149127 | 149142 | T | C | WS1 234 | M153R: L204S |
| 149127 | 149155 | T | C | WS6 1071 | M153R: L204S |
| 149127 | 149000 | T | C | WS1 328 | M153R: L204S |
| 149127 | 148971 | T | C | WS6 346 | M153R: L204S |
| 149127 | 149198 | T | C | SWH 8-2-93 | M153R: L204S |
| 149127 | 149810 | T | C | SWH 1209 | M153R: L204S |
| 149127 | 149120 | T | C | Glenfield | M153R: L204S |
| 149127 | 149064 | T | C | BRK | M153R: L204S |
| 149127 | 149160 | T | C | SWH | M153R: L204S |
| 149127 | 148991 | T | C | Meby | M153R: L204S |
| 149127 | 149129 | T | C | Bendigo | M153R: L204S |
| 149127 | 149133 | T | C | KM13 | M153R: L204S |
| 149127 | 149130 | T | C | Uriarra | M153R: L204S |
| 149223 | 149161 | - | T | BRK | intergenic |
| 149223 | 149257 | - | T | SWH | intergenic |
| 149223 | 149156 | - | T | OB1 406 | intergenic |
| 149223 | 149157 | - | T | OB1 406 | intergenic |
| 149223 | 149097 | - | T | WS1 328 | intergenic |
| 149223 | 149225 | - | T | SWH 8-2-93 | intergenic |
| 149512 | 149376 | G | A | Meby | M154L: synonymous |
| 149605 | 149539 | C | T | OB1 406 | M154L: synonymous |
| 149605 | 149479 | C | T | WS1 328 | M154L: synonymous |
| 149605 | 149543 | C | T | BRK | M154L: synonymous |
| 149605 | 149639 | C | T | SWH | M154L: synonymous |
| 149629 | 149632 | C | A | Uriarra | M154L: synonymous |
| 149717 | 149588 | T | C | BRK 12-2-93 | M154L: Y53C |
| 149717 | 149694 | T | C | Wellington | M154L: Y53C |
| 149717 | 149627 | T | C | BRK 897 | M154L: Y53C |
| 149717 | 149651 | T | C | OB1 406 | M154L: Y53C |
| 149717 | 149732 | T | C | WS1 234 | M154L: Y53C |
| 149717 | 149745 | T | C | WS6 1071 | M154L: Y53C |
| 149717 | 149591 | T | C | WS1 328 | M154L: Y53C |
| 149717 | 149561 | T | C | WS6 346 | M154L: Y53C |
| 149717 | 149719 | T | C | SWH 8-2-93 | M154L: Y53C |
| 149717 | 149655 | T | C | BRK | M154L: Y53C |
| 149717 | 149751 | T | C | SWH | M154L: Y53C |
| 149717 | 150146 | T | C | BD23 | M154L: Y53C |
| 149717 | 150400 | T | C | SWH 1209 | M154L: Y53C |
| 149717 | 149719 | T | C | Bendigo | M154L: Y53C |
| 149836 | 149707 | G | A | BRK 12-2-93 | M154L: synonymous |
| 149836 | 149770 | G | A | OB1 406 | M154L: synonymous |
| 149836 | 149864 | G | A | WS6 1071 | M154L: synonymous |
| 149836 | 149710 | G | A | WS1 328 | M154L: synonymous |
| 149836 | 149680 | G | A | WS6 346 | M154L: synonymous |
| 149836 | 149774 | G | A | BRK | M154L: synonymous |
| 149836 | 149870 | G | A | SWH | M154L: synonymous |
| 149836 | 149838 | G | A | Bendigo | M154L: synonymous |
| 149836 | 150265 | G | A | BD23 | M154L: synonymous |
| 149836 | 150519 | G | A | SWH 1209 | M154L: synonymous |
| 149907 | 149934 | T | - | WS6 1071 | intergenic |
| 149907 | 149885 | - | T | Wellington | intergenic |
| 149907 | 149818 | - | T | BRK 897 | intergenic |
| 149907 | 149923 | - | T | WS1 234 | intergenic |
| 149907 | 149910 | - | T | SWH 8-2-93 | intergenic |
| 149917 | 149828 | G | C | BRK 897 | intergenic |
| 149917 | 149933 | G | C | WS1 234 | intergenic |
| 150143 | 150007 | C | T | Meby | M156R: synonymous |
| 150280 | 150151 | T | C | BRK 12-2-93 | M156R: L98P |
| 150280 | 150258 | T | C | Wellington | M156R: L98P |
| 150280 | 150191 | T | C | BRK 897 | M156R: L98P |
| 150280 | 150296 | T | C | WS1 234 | M156R: L98P |
| 150280 | 150307 | T | C | WS6 1071 | M156R: L98P |
| 150280 | 150124 | T | C | WS6 346 | M156R: L98P |
| 150280 | 150709 | T | C | BD23 | M156R: L98P |
| 150280 | 150963 | T | C | SWH 1209 | M156R: L98P |
| 150287 | 150130 | T | - | WS6 346 | M156R: readthrough |
| 150915 | 150758 | G | A | WS6 346 | M008.1: synonymous |
| 151453 | 151317 | C | T | Meby | M008.1: synonymous |
| 152643 | 152486 | C | T | WS6 346 | M008R: A418V |
| 152776 | 152714 | C | T | BRK | M008R: synonymous |
| 152776 | 152810 | C | T | SWH | M008R: synonymous |
| 152776 | 152710 | C | T | OB1 406 | M008R: synonymous |
| 152776 | 153205 | C | T | BD23 | M008R: synonymous |
| 152776 | 153459 | C | T | SWH 1209 | M008R: synonymous |
| 152776 | 152779 | C | T | SWH 8-2-93 | M008R: synonymous |
| 152776 | 152650 | C | T | WS1 328 | M008R: synonymous |
| 152776 | 152778 | C | T | Bendigo | M008R: synonymous |
| 153275 | 153302 | C | T | WS6 1071 | M007R: A96V |
| 153354 | 153381 | C | T | WS6 1071 | M007R: synonymous |
| 154009 | 153880 | A | C | BRK 12-2-93 | M006R: T65P |
| 154047 | 154730 | A | G | SWH 1209 | M006R: I77M |
| 154328 | 154185 | A | G | Gung | M006R: D171G |
| 155411 | 155415 | - | C | Uriarra | M005R: frameshift |
| 156008 | 156042 | C | A | SWH | M005R: S209Y |
| 156008 | 155872 | C | A | Meby | M005R: S209Y |
| 156008 | 156010 | C | A | Bendigo | M005R: S209Y |
| 156008 | 155946 | C | A | BRK | M005R: S209Y |
| 156008 | 155865 | C | A | Gung | M005R: S209Y |
| 156008 | 155882 | C | A | WS1 328 | M005R: S209Y |
| 156008 | 155851 | C | A | WS6 346 | M005R: S209Y |
| 156008 | 155919 | C | A | BRK 897 | M005R: S209Y |
| 156008 | 155942 | C | A | OB1 406 | M005R: S209Y |
| 156008 | 156035 | C | A | WS6 1071 | M005R: S209Y |
| 156008 | 156437 | C | A | BD23 | M005R: S209Y |
| 156008 | 156691 | C | A | SWH 1209 | M005R: S209Y |
| 156008 | 155986 | C | A | Wellington | M005R: S209Y |
| 156008 | 156011 | C | A | SWH 8-2-93 | M005R: S209Y |
| 156008 | 156024 | C | A | WS1 234 | M005R: S209Y |
| 156008 | 155879 | C | A | BRK 12-2-93 | M005R: S209Y |
| 156048 | 155922 | A | G | WS1 328 | M005R: synonymous |
| 156230 | 156074 | - | G | WS6 346 | M005R: frameshift |
| 156582 | 156609 | C | T | WS6 1071 | M005R: synonymous |
| 156682 | 156620 | C | T | BRK | M005R: R434W |
| 156682 | 156716 | C | T | SWH | M005R: R434W |
| 156682 | 156546 | C | T | Meby | M005R: R434W |
| 156682 | 156539 | C | T | Gung | M005R: R434W |
| 156682 | 156556 | C | T | WS1 328 | M005R: R434W |
| 156682 | 156526 | C | T | WS6 346 | M005R: R434W |
| 156682 | 156593 | C | T | BRK 897 | M005R: R434W |
| 156682 | 156616 | C | T | OB1 406 | M005R: R434W |
| 156682 | 156709 | C | T | WS6 1071 | M005R: R434W |
| 156682 | 157111 | C | T | BD23 | M005R: R434W |
| 156682 | 157365 | C | T | SWH 1209 | M005R: R434W |
| 156682 | 156660 | C | T | Wellington | M005R: R434W |
| 156682 | 156684 | C | T | Bendigo | M005R: R434W |
| 156682 | 156685 | C | T | SWH 8-2-93 | M005R: R434W |
| 156682 | 156998 | C | T | WS1 234 | M005R: R434W |
| 156682 | 156553 | C | T | BRK 12-2-93 | M005R: R434W |
| 157543 | 157407 | C | G | Meby | M004R: N138K |
| 157588 | 157445 | G | A | Gung | M004R; synonymous |
| 157588 | 157615 | G | A | WS6 1071 | M004R; synonymous |
| 157808 | 157842 | C | T | SWH | M004R: P227S |
| 157908 | 158592 | T | - | SWH 1209 | intergenic |
| 158596 | 158534 | C | T | BRK | M003.1R: A37V |
| 158596 | 158630 | C | T | SWH | M003.1R: A37V |
| 158596 | 158460 | C | T | Meby | M003.1R: A37V |
| 158596 | 158598 | C | T | Bendigo | M003.1R: A37V |
| 158596 | 158453 | C | T | Gung | M003.1R: A37V |
| 158596 | 158470 | C | T | WS1 328 | M003.1R: A37V |
| 158596 | 158440 | C | T | WS6 346 | M003.1R: A37V |
| 158596 | 158507 | C | T | BRK 897 | M003.1R: A37V |
| 158596 | 158530 | C | T | OB1 406 | M003.1R: A37V |
| 158596 | 158623 | C | T | WS6 1071 | M003.1R: A37V |
| 158596 | 159025 | C | T | BD23 | M003.1R: A37V |
| 158596 | 159278 | C | T | SWH 1209 | M003.1R: A37V |
| 158596 | 158574 | C | T | Wellington | M003.1R: A37V |
| 158596 | 158599 | C | T | SWH 8-2-93 | M003.1R: A37V |
| 158596 | 158612 | C | T | WS1 234 | M003.1R: A37V |
| 158596 | 158467 | C | T | BRK 12-2-93 | M003.1R: A37V |
| 158712 | 158714 | C | G | Bendigo | M003.1R: L76V |
| 158712 | 158690 | C | G | Wellington | M003.1R: L76V |
| 158712 | 158623 | C | G | BRK 897 | M003.1R: L76V |
| 158712 | 158715 | C | G | SWH 8-2-93 | M003.1R: L76V |
| 158712 | 158728 | C | G | WS1 234 | M003.1R: L76V |
| 158733 | 158760 | C | T | WS6 1071 | M003.1R: H83Y |
| 158940 | 158941 |  | 27 nt del | Bendigo | intergenic |
| 158940 | 158917 |  | 27 nt del | Wellington | intergenic |
| 158940 | 158796 |  | 27 nt del | Gung | intergenic |
| 158940 | 158850 |  | 27 nt del | BRK 897 | intergenic |
| 158940 | 158942 |  | 27 nt del | SWH 8-2-93 | intergenic |
| 158940 | 158955 |  | 27 nt del | WS1 234 | intergenic |
| 158940 | 158783 |  | 27 nt del | WS6 346 | intergenic |
| 158981 | 158797 |  | 12 nt del | WS6 346 | intergenic |
| 159140 | 159078 | A | G | BRK | M002R: synonymous |
| 159140 | 159174 | A | G | SWH | M002R: synonymous |
| 159140 | 159074 | A | G | OB1 406 | M002R: synonymous |
| 159140 | 159014 | A | G | WS1 328 | M002R: synonymous |
| 159188 | 159018 | A | G | Gung | M002R: synonymous |
| 159188 | 159059 | A | G | BRK 12-2-93 | M002R: synonymous |
| 159188 | 159139 | A | G | Wellington | M002R: synonymous |
| 159188 | 159617 | A | G | BD23 | M002R: synonymous |
| 159188 | 159072 | A | G | BRK 897 | M002R: synonymous |
| 159188 | 159122 | A | G | OB1 406 | M002R: synonymous |
| 159188 | 159177 | A | G | WS1 234 | M002R: synonymous |
| 159188 | 159215 | A | G | WS6 1071 | M002R: synonymous |
| 159188 | 159062 | A | G | WS1 328 | M002R: synonymous |
| 159188 | 158993 | A | G | WS6 346 | M002R: synonymous |
| 159188 | 159164 | A | G | SWH 8-2-93 | M002R: synonymous |
| 159188 | 159870 | A | G | SWH 1209 | M002R: synonymous |
| 159188 | 159181 | A | G | Glenfield | M002R: synonymous |
| 159188 | 159126 | A | G | BRK | M002R: synonymous |
| 159188 | 159222 | A | G | SWH | M002R: synonymous |
| 159188 | 159052 | A | G | Meby | M002R: synonymous |
| 159188 | 159163 | A | G | Bendigo | M002R: synonymous |
| 159188 | 159194 | A | G | KM13 | M002R: synonymous |
| 159188 | 159192 | A | G | Uriarra | M002R: synonymous |
| 159469 | 159444 | A | G | Bendigo | M002R: Q117R |
| 159469 | 159299 | A | G | Gung | M002R: Q117R |
| 159681 | 159511 | A | G | Gung | M002R: T188A |
| 159796 | 159734 | C | T | BRK | M002R: A226V |
| 159796 | 159830 | C | T | SWH | M002R: A226V |
| 159796 | 159660 | C | T | Meby | M002R: A226V |
| 159796 | 159771 | C | T | Bendigo | M002R: A226V |
| 159796 | 158626 | C | T | Gung | M002R: A226V |
| 159796 | 159670 | C | T | WS1 328 | M002R: A226V |
| 159796 | 159601 | C | T | WS6 346 | M002R: A226V |
| 159796 | 159680 | C | T | BRK 897 | M002R: A226V |
| 159796 | 159730 | C | T | OB1 406 | M002R: A226V |
| 159796 | 159823 | C | T | WS6 1071 | M002R: A226V |
| 159796 | 160478 | C | T | SWH 1209 | M002R: A226V |
| 159796 | 160225 | C | T | BD23 | M002R: A226V |
| 159796 | 159747 | C | T | Wellington | M002R: A226V |
| 159796 | 159772 | C | T | SWH 8-2-93 | M002R: A226V |
| 159796 | 159785 | C | T | WS1 234 | M002R: A226V |
| 159796 | 159667 | C | T | BRK 12-2-93 | M002R: A226V |
| 159977 | 159848 | G | A | BRK 12-2-93 | M002R: synonymous |
| 160129 | 160081 | - | A | Wellington | intergenic |
| 160385 | 160256 | G | A | BRK 12-2-93 | M001R: G69D |
| 160817 | 160769 | G | A | Wellington | M001R: S213N |
| 160817 | 160701 | G | A | BRK 897 | M001R: S213N |
| 160817 | 160806 | G | A | WS1 234 | M001R: S213N |
| 161004 | 160834 | G | A | Gung | intergenic |
| 161059 | 160944 | - | T | BRK 897 | intergenic |
| 161059 | 161049 | - | T | WS1 234 | intergenic |
| 161060 | 160889 | T | - | Gung | intergenic |
| 161060 | 161741 | T | - | SWH 1209 | intergenic |
| 161182 | 161011 | C | T | Gung | intergenic |
| 161264 | 161149 | C | T | BRK 897 | M000.5R: synonymous |
| 161264 | 161254 | C | T | WS1 234 | M000.5R: synonymous |
| 161601 | 162282 | G | A | SWH 1209 | non-coding |
| 161601 | 161628 | G | A | WS6 1071 | non-coding |
| 161616 | 161642 | C | - | WS6 1071 | non-coding |
| 161623 | 161507 | C | - | BRK 897 | non-coding |
| 161653 | 161536 | A | - | BRK 897 | non-coding |
| 161668 | 161606 | T | G | BRK | non-coding |
| 161692 | 161719 | - | A | WS6 1071 | non-coding |
| 161710 | 161738 | - | C | WS6 1072 | non-coding |
| 161740 | 161747 | - | T | KM13 | non-coding |
| 161742 | 161750 | - | T | KM13 | non-coding |
| 161742 | 161747 | - | T | Uriarra | non-coding |
| 161742 | 161719 | - | T | SWH 8-2-93 | non-coding |
| 161742 | 161733 | - | T | WS1 234 | non-coding |
