# Supplementary material for: Evolutionary History and Attenuation of Myxoma Virus on Two Continents
Source: PLoS Pathog. 2012 Oct 4;8(10):e1002950. doi: 10.1371/journal.ppat.1002950 (PMC3464225; doi:10.1371/journal.ppat.1002950)
Supplement: Table S2 — Sequence differences between Lu and the European isolates of MYXV sequenced here. (DOC) [file ppat.1002950.s002.doc]

**Table S2**. Sequence differences between Lu and the European isolates of MYXV sequenced here.

*Also referred to as Nottingham attenuated

| **Lu position** | **Sequence position** | **Lu sequence** | **Mutation** | **Virus** | **Gene: change** |
| --- | --- | --- | --- | --- | --- |
| 22 | 21 | A | - | Nott* | non-coding |
| 22 | 21 | A | - | Cornwall | non-coding |
| 2698 | 2697 | G | A | Nott | intergenic |
| 3856 | 3854 | A | - | Nott | intergenic |
| 15761 | 15759 | C | T | Nott | M014L: E269K |
| 20489 | 20487 | C | T | Nott | M020L: E16K |
| 21130 | 21129 | T | C | Cornwall | M021L: D467G |
| 33025 | 33024 | C | T | Cornwall | M032R: T565I |
| 37955 | 37954 | - | T | Nott | M036L: frameshift |
| 47414 | 47413 | C | T | Cornwall | M045L: synonymous |
| 82925 | 82924 | G | A | Cornwall | M084R: synonymous |
| 108648 | 108647 | G | A | Nott | M113R: synonymous |
| 128422 | 128421 | G | A | Nott | M134R: D909N |
| 131607 | 131607 | - | A | Nott | M134R: frameshift |
| 134073 | 134073 | G | A | Nott | M138L: synonymous |
| 140040 | 140040 | C | T | Nott | M144R: A209V |
| 140040 | 140039 | C | T | Cornwall | M144R: A209V |
| 142906 | 142905 | T | C | Cornwall | M148R: V426A |
| 145755 | 145756 | - | T | Nott | M150R: frameshift |
| 145755 | 145757 | - | G | Nott | M150R: frameshift |
| 147617 | 147619 | C | T | Nott | M151R: synonymous |
| 148166 | 148165 | A | G | Cornwall | M152R: S159G |
| 157922 | 157923 | T | - | Nott | intergenic |
| 159080 | 159081 | C | T | Nott | intergenic |
| 161756 | 161756 | T | - | Nott | non-coding |
| 161756 | 161754 | T | - | Cornwall | non-coding |
